# Liver function differences in atherosclerotic cardiovascular disease: a multi-ethnic dual-cohort retrospective study

**DOI:** 10.3389/fendo.2025.1558872

**Published:** 2025-03-24

**Authors:** Yifei Wang, Zichen Zhang, Wenbo Ren, Lin Shi, Taiyu Zhai, Jing Huang

**Affiliations:** ^1^ Department of Clinical Laboratory, The First Hospital of Jilin University, Changchun, China; ^2^ College of Medical Technology, Beihua University, Jilin, China

**Keywords:** atherosclerotic cardiovascular disease, liver function, risk assessment, hepatocellular injury indicators, metabolic function

## Abstract

**Background and aims:**

Liver function plays a pivotal role in the initiation and progression of atherosclerotic cardiovascular disease (ASCVD). Exploring the potential associations between liver function assessment indicators and ASCVD is essential for understanding the liver’s involvement in ASCVD pathogenesis. However, the specific relationships between these indicators and ASCVD are still debated. This study aims to conduct an in-depth comparative analysis of variations in various liver function assessment indicators among populations of ASCVD patients.

**Methods:**

A dual-cohort retrospective cross-sectional study design was employed, using data from 15,943 ASCVD patients at the First Hospital of Jilin University and 472 ASCVD patients from the National Health and Nutrition Examination Survey (NHANES) database. Liver function indicators, including enzymatic, protein synthesis, bilirubin metabolism indices, and lipid profile parameters, were analyzed. Inclusion and exclusion criteria were rigorously applied, followed by univariate regression, multivariate regression and stratified subgroup analyses.

**Results:**

Hepatocyte damage indicators (aspartate aminotransferase, alanine aminotransferase, gamma-glutamyl transferase, alkaline phosphatase) and total bilirubin were identified as risk factors for ASCVD. Albumin showed a protective effect. Globulin levels differed significantly between cohorts. Cholinesterase (cohort 1) and total protein, total cholesterol (cohort 2) showed no significant changes in ASCVD patients.

**Conclusion:**

Many liver function indicators are correlated with ASCVD. There are differences in these indicators between ASCVD patients and healthy volunteers. Although some indicators may be weakly correlated due to confounding factors, this study still provides a scientific rationale for developing more precise ASCVD prevention and treatment strategies in the future.

## 1 Introduction

Atherosclerotic cardiovascular disease (ASCVD), a prevalent type of cardiovascular disease (CVD), has consistently witnessed an increase in morbidity and mortality globally, attracting widespread attention (1). In recent years, researchers have conducted in-depth explorations of the pathogenesis of ASCVD, emphasizing that the assessment of liver function-related indicators is crucial for uncovering potential links between liver diseases and ASCVD (2). This finding further underscores the crucial role of liver function in the initiation and progression of ASCVD.

Liver function assessment indicators are generally categorized into: Enzymatic markers, including aspartate aminotransferase (AST), alanine aminotransferase (ALT), gamma-glutamyl transferase (GGT), and alkaline phosphatase (ALP). Protein synthesis indicators, including albumin (ALB), globulin (GLO), and the albumin-to-globulin ratio (A/G). Bilirubin metabolism indicators, including total bilirubin (TBIL), direct bilirubin (DBIL), and indirect bilirubin (IBIL). Other lipid-related parameters, including high-density lipoprotein cholesterol (HDL-C), low-density lipoprotein cholesterol (LDL-C), and apolipoprotein B (ApoB).

In the past few decades, liver function assessment indicators have attracted considerable attention and sparked extensive discussions as potential risk markers for ASCVD. However, despite the widespread mention of potential associations between these indicators and ASCVD, the underlying mechanisms linking them remain controversial. Against this backdrop, research into the relationship between liver function assessment indicators and the incidence and mortality rates of ASCVD has continued to intensify, and resulting in significant advancements. Yet, the results of these studies have exhibited diverse trends, with some providing evidence of positive correlations, while others are contradictory or conditionally correlated. This further underscores the complexity of this field and the necessity for in-depth research (3–5).

For instance, regarding liver enzyme indicators, studies have pointed out that elevated levels of GGT are closely associated with an increased risk of ASCVD, often regarded as a marker of heightened ASCVD risk (2, 6). Furthermore, when considering protein synthesis indicators, an increase in serum ALB concentration has been linked to a reduction in mortality among ASCVD patients, while GLO has also been identified as an independent covariate influencing ASCVD mortality (7, 8). Additionally, in terms of bilirubin metabolism indicators, research suggests that a mild elevation in serum bilirubin levels may be associated with a decreased risk of ASCVD (9, 10). Lastly, regarding other lipid indicators, there is an inverse relationship between HDL-C levels and ASCVD risk. Importantly, ApoB and lipoprotein(a) (Lp(a)) may play more pivotal roles in ASCVD risk assessment compared to LDL-C, further strengthening the association between these lipid indicators and ASCVD (11–13).

However, it is worth noting that not all studies consistently corroborate these associations. For instance, in terms of liver enzyme indicators, other studies have demonstrated that incorporating GGT into ASCVD risk assessment exhibits limited predictive value for first adverse cardiovascular events (14). Similarly, regarding bilirubin metabolism indicators, studies have indicated that the addition of TBIL information to traditional risk factors does not substantially enhance the predictive efficacy for ASCVD risk (15). These findings imply that, despite the notable associations between liver function assessment indicators and ASCVD incidence or mortality, there remains a pressing need for more credible and direct evidence to elucidate the specific mechanisms and the precise magnitude of these associations.

Given the existing controversies and deficiencies in current research regarding the associations between liver function assessment indicators and ASCVD, this study aims to maximize the utilization of extensive clinical data resources from the First Hospital of Jilin University, augmented by information from the National Health and Nutrition Examination Survey (NHANES) database, leveraging real-world testing data. Utilizing a large-sample, multi-ethnic, two-cohort retrospective cross-sectional study design, this study intends to conduct a comprehensive comparative analysis of the manifestations of liver function assessment indicators in ASCVD patients. Furthermore, the objective is to further validate and elucidate potential correlations between these indicators and ASCVD, thereby providing more compelling evidence to address the inconsistencies and controversies in existing research. Additionally, this endeavor not only seeks to offer valuable insights into the clinical application of liver function assessment indicators in non-traditional liver disease areas but also aims to illuminate new pathways in understanding the pathogenesis of ASCVD.

## 2 Materials and methods

### 2.1 Inclusion and exclusion criteria for study subjects

This research endeavor integrates clinical data sourced from the First Hospital of Jilin University in China with information from the NHANES database in the United States to explore the potential associations between liver function assessment indicators and related laboratory biochemical markers with ASCVD. Ethical approval for this study was granted by the Institutional Review Board and Ethics Committee of the First Hospital of Jilin University, under Approval Number 2024-729, and the research was conducted in strict adherence to the ethical guidelines outlined in the Helsinki Declaration. For cohort 1, data were collected from ASCVD patients who sought treatment at the First Hospital of Jilin University between September 1, 2022, and June 1, 2024, utilizing the hospital’s information management system. The inclusion criteria for the ASCVD patient cohort encompassed individuals with a clinical diagnosis of coronary heart disease, coronary atherosclerotic heart disease, angina pectoris, or myocardial infarction, who were experiencing their first ASCVD event and lacked a prior treatment history. Data from patients with signs of lipemia or hemolysis, unclear diagnoses, or from those suffering from other diseases were excluded. For the control group, healthy volunteers with all indicators falling within the normal reference range were recruited from the physical examination center (The normal reference value ranges utilized for screening these volunteers are detailed in **Supplementary Table 1**).

Cohort 2 data were gathered from the NHANES database, specifically utilizing follow-up information spanning from 2017 to 2020. During the initial screening phase, individuals with liver and other diseases and those lacking essential liver function test indicators were excluded. Subsequently, subject samples with examination indicators outside the normal reference range were omitted and not considered for inclusion in the control group of this study. To ascertain ASCVD status among participants, questionnaire responses were rigorously examined. Participants who affirmatively responded to questions related to coronary heart disease, angina pectoris, or stroke were categorized as having ASCVD and grouped accordingly. The laboratory results utilized in this analysis were derived from serum specimens collected at mobile examination centers and were processed by the Advanced Research Diagnostics Laboratory (ARDL) at the University of Minnesota (The normal reference value ranges utilized for screening are detailed in **Supplementary Table 1**).

### 2.2 Assessment of covariates

Due to the lack of height, weight, and lifestyle data in the hospital’s information management system, we have not yet collected disease-related covariates for inclusion in Study Cohort 1. However, following preliminary literature research, we have introduced a series of covariates associated with ASCVD in Study Cohort 2, including BMI, smoking status, alcohol consumption, diabetes, and hypertension, all of which were identified based on literature (16, 17). BMI was calculated by dividing body weight (kg) by the square of height (m²). Smoking status was determined based on participants’ responses of ‘yes’ or ‘no’ to the question ‘Do you now smoke cigarettes?’ in the questionnaire. Recent alcohol use status was assessed according to the definition of recent alcohol use in the Dietary Guidelines for Americans (2020–2025), with participants selecting ‘yes’ or ‘no’ to the question ‘Ever have 4/5 or more drinks every day?’ (male = 5, female = 4) in the questionnaire (U.S. Department of Health and Human Services and U.S. Department of Agriculture (2020). Dietary Guidelines for Americans, 2020-2025. Retrieved from https://odphp.health.gov/our-work/nutrition-physical-activity/dietary-guidelines/current-dietary-guidelines). Diabetes and hypertension were determined based on self-reported physician diagnoses from the questionnaire. These indicators, serving as covariates, facilitate better observation of the potential correlations between ASCVD and various liver function indicators.

### 2.3 Data analysis

All data analysis in this full-text study was conducted on the DxAI platform (https://www.xsmartanalysis.com). We used R version 4.2.3 and the gtsummary package version 1.7.2 for all descriptive statistics and baseline data analysis. Additionally, the statsmodels Python package, version 0.11.1, was utilized for univariate regression analysis, multifactorial regression analysis and hierarchical regression analysis.

Among these analyses, descriptive statistics encompass summarizing categorical data, such as gender and group, as well as summarizing data related to age and various biochemical indicators, incorporating counts or categories where appropriate. This involves presenting information on the frequency, mean, extreme values, standard deviation, and rates of missing data for the variables. The aim is to furnish an overview of the data distribution and characteristics of the variables, thereby aiding in the understanding of the sample population and the data being analyzed.

For Cohort I baseline analysis, the group was designated as the grouping variable, with gender and age serving as categorical variables, and biochemical indicators acting as quantitative variables. For Cohort II baseline analysis, the group was similarly designated as the grouping variable, while gender, age, recent alcohol use status, diabetes, and hypertension were considered categorical variables, with BMI and biochemical indicators continuing to be quantitative variables. Based on sample characteristics, including the number of groups, sample type, sample size, normality, and homogeneity of variances, appropriate statistical methodologies are chosen to produce a statistical results table that consists of three rows of data. The specific approach is as follows: Compare the data between the ASCVD group and the control group. For categorical variables, the Chi-square test is used; for quantitative variables that do not follow a normal distribution, the Mann-Whitney U test is applied. The final table presents information such as the mean, standard deviation, missing data points, and P-values. Furthermore, we used GraphPad Prism version 9.5.0 software to create bar charts illustrating the differences, which visually depict the aforementioned information and highlight the disparities in various indicators across different groups.

In the univariate regression analysis, Cohort I set the group as the dependent variable, age and gender as categorical independent variables, and various biochemical indicators as quantitative independent variables. Cohort II adjusted the analysis based on Cohort I, incorporating BMI, recent alcohol use status, diabetes, and hypertension into consideration. Using a logistic regression model, tables and forest plots were obtained, containing frequency, odds ratio (OR) values, 95% confidence intervals (CIs), and P-values. In stratified regression analysis for subgroups, we designated the presence or absence of ASCVD in patients as the dependent variable, other tested indicators as exposure variables, and gender and age as stratification variables. Applying the logistic regression model and excluding biochemical indicators that were not statistically significant in the univariate regression analysis, we derived a series of forest plots encompassing frequency, OR values, 95% CI, and P-values. These forest plots offer a comprehensive visualization of the associations between the exposure variables and ASCVD risk, stratified by gender and age. Also, we calculated E-values in sensitivity analyses to assess the potential impact of unmeasured confounders on study outcomes (18).

### 2.4 Statistical analyses

The data are presented as means and standard deviations (SDs) for each group. The Chi-square test was utilized to compare the gender distribution between the control group and the ASCVD group, whereas the Mann-Whitney U test was applied for comparisons of other indicators. When comparing data across multiple groups, we first used ANOVA; if significant differences were detected, we conducted more detailed pairwise comparisons using Fisher’s Least Significant Difference (LSD) test. A P-value of less than 0.05 was considered statistically significant.

## 3 Results

### 3.1 Descriptive statistic

**Figure 1:**
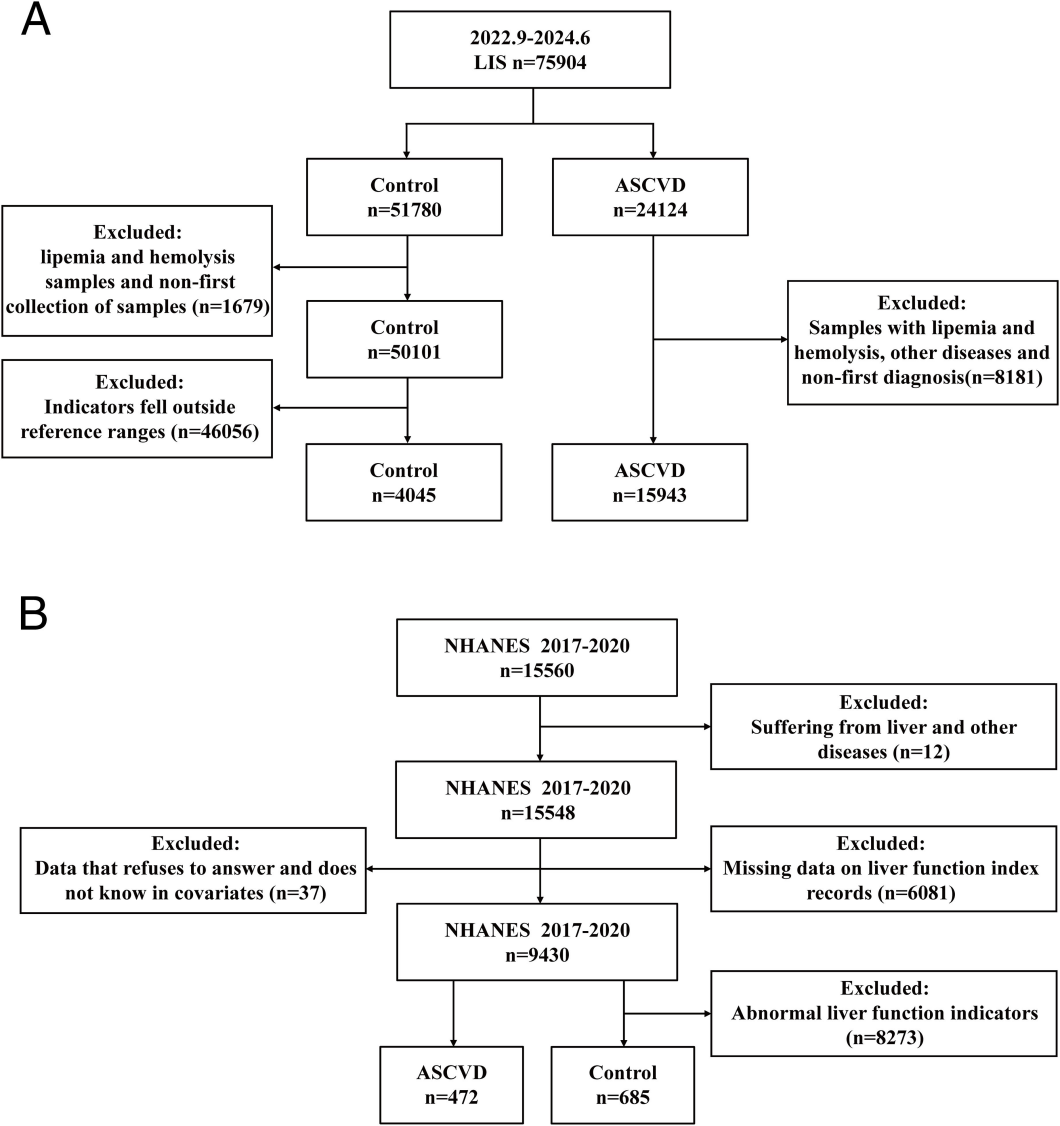
Detailed illustration of sample inclusion criteria and grouping procedures. **(A)** Inclusion and exclusion process for cohort 1 (sourced from the First Hospital of Jilin University): Following initial screening, a total of 15,943 samples were identified and included in the ASCVD group, while 4,045 samples were identified and included in the control group. **(B)** Inclusion and exclusion process for cohort 2 (sourced from the NHANES database): After the initial screening stage, 472 samples were selected and included in the ASCVD group, and 685 samples were selected and included in the control group.

The data for cohort 1 were sourced from the First Hospital of Jilin University. A total of 24,124 ASCVD patients who visited the hospital between September 1, 2022, and June 1, 2024, were recorded. After excluding 8,181 samples due to hemolysis, lipemia, other diseases and non-first-time diagnoses, 15,943 samples with ASCVD were ultimately included as the ASCVD group. Additionally, 51,780 samples from the hospital’s physical examination center during the same period were collected. After excluding 1,679 samples due to hemolysis, lipemia, and non-first-time collections, and further excluding 46,056 samples with liver function indicators not within the reference range, 4,045 samples were ultimately included as the control group (**Figure 1A**). Among these samples, the ASCVD group and males had the highest frequency of a certain condition or characteristic (**Supplementary Table 2**). Among all the obtained liver function indicators, triglyceride (TG), HDL-C, and LDL-C had no data available in the control group; and the missing rates for apolipoprotein A-I(ApoA1), ApoB, Lp(a), and Homocysteine (HCY) exceeded 90%. Therefore, these indicators will not be included in subsequent research (**Supplementary Table 3**).

The data for cohort 2 were sourced from the NHANES database. A total of 15,560 follow-up records from 2017 to 2020 were collected. Initially, 12 patient records with common liver and other diseases were excluded. Subsequently, 6,081 samples with missing liver function indicator data were excluded. At the same time, data from 37 samples where the above covariates showed refusal to answer and don’t know in the questionnaire answers were also excluded. Finally, 8,273 samples from the control group with liver function indicators not within the healthy reference range were excluded, while 472 patient records with ASCVD were included as the ASCVD group. The final samples included in the study consisted of 472 ASCVD cases and 685 controls (**Figure 1B**). All participants provided information on age, gender, health status, and other relevant factors through questionnaires. Among these participants, the control group and males had the highest frequency of a certain characteristic or condition (**Supplementary Table 4**). Among all the obtained data, LDL-C had a missing rate of 57.822%, smoking status had a missing rate of 54.192% will not be included in subsequent research (**Supplementary Tables 4**, **5**).

### 3.2 Baseline analysis

When comparing the baseline characteristics between the ASCVD group and the control group, we conducted a comprehensive analysis to ascertain the overall differences between the two. The results demonstrated significant disparities in several critical indicators across the two groups.

**Figure 2:**
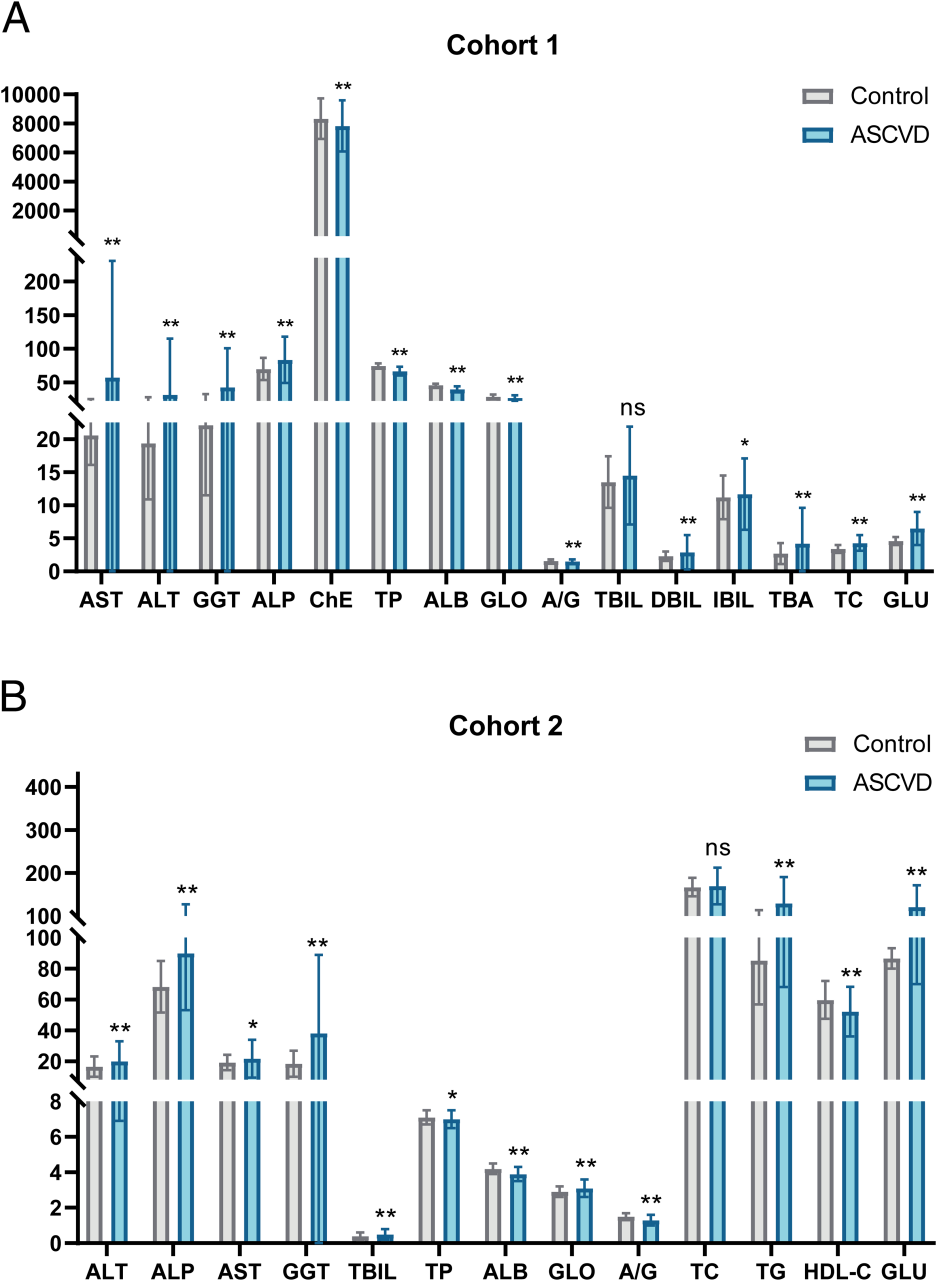
Significant differences in multiple indicators between control and ASCVD groups in two cohorts. **(A)** Visual bar chart of baseline analysis data for cohort 1. **(B)** Visual bar chart of baseline analysis data for cohort 2. Gray bars represent the control group, while blue bars signify the ASCVD group. The line segments above the bars indicate the standard deviation (SD) for the respective group’s data. Significance levels are denoted as follows: “**” indicates a P-value ≤ 0.01, “*” indicates a P-value ≤ 0.05, and “ns” indicates a P-value greater than 0.05.

In cohort 1, the ASCVD group exhibited significantly elevated mean levels of various biochemical indicators compared to the control group, including AST (57.3 ± 173.2), ALT (31.8 ± 83.2), GGT (42.8 ± 58.1), ALP (83.7 ± 34.4), DBIL (2.9 ± 2.6), Total biliary acid (TBA) (4.2 ± 5.4), TC (4.3 ± 1.2), and glucose (GLU) (6.5 ± 2.5) (**Table 1**). Conversely, the ASCVD group had lower levels of cholinesterase (ChE), total protein (TP), ALB, GLO, and A/G compared to the control group (**Figure 2A**). Notably, TBIL did not show a significant difference between the two groups in cohort 1.

**Table 1:** Baseline characteristics of participants in two cohorts.

Var	Cohort 1			Cohort 2		
	Control N = 4,045	ASCVD N = 15,943	p^a^	Control N = 685	ASCVD N = 472	p^a^
**AST**			<0.001			0.043
Mean (SD)	20.6 (4.5)	57.3 (173.2)		19.2 (5.0)	21.6 (12.3)	
**ALT**			<0.001			<0.001
Mean (SD)	19.4 (8.5)	31.8 (83.2)		16.5 (6.6)	19.9 (13.0)	
**GGT**			<0.001			<0.001
Mean (SD)	22.1 (10.6)	42.8 (58.1)		18.4 (8.4)	38.0 (50.9)	
**ALP**			<0.001			<0.001
Mean (SD)	69.9 (16.4)	83.7 (34.4)		68.2 (16.7)	90.0 (36.9)	
**ChE**			<0.001			NA
Mean (SD)	8,328.8 (1,400.0)	7,836.3 (1,764.0)		NA	NA	
**TP**			<0.001			0.017
Mean (SD)	74.8 (3.5)	66.7 (6.4)		7.1 (0.4)	7.0 (0.5)	
**ALB**			<0.001			<0.001
Mean (SD)	45.9 (2.4)	39.8 (4.5)		4.2 (0.3)	3.9 (0.4)	
**GLO**			<0.001			<0.001
Mean (SD)	28.9 (3.0)	26.9 (4.1)		2.9 (0.3)	3.1 (0.5)	
**A/G**			<0.001			<0.001
Mean (SD)	1.6 (0.2)	1.5 (0.3)		1.5 (0.2)	1.3 (0.3)	
**TBIL**			0.12			<0.001
Mean (SD)	13.5 (3.9)	14.5 (7.4)		0.4 (0.2)	0.5 (0.3)	
**DBIL**			<0.001			NA
Mean (SD)	2.3 (0.7)	2.9 (2.6)		NA	NA	
**IBIL**			0.007			NA
Mean (SD)	11.2 (3.3)	11.7 (5.4)		NA	NA	
**TBA**			<0.001			NA
Mean (SD)	2.7 (1.6)	4.2 (5.4)		NA	NA	
**TC**			<0.001			0.357
Mean (SD)	3.4 (0.6)	4.3 (1.2)		167.1 (21.7)	170.0 (42.6)	
**TG**			NA			<0.001
Mean (SD)	NA	NA		85.3 (28.5)	129.3 (61.3)	
**HDL-C**			NA			<0.001
Mean (SD)	NA	NA		59.7 (12.2)	52.1 (16.1)	
**GLU**			<0.001			<0.001
Mean (SD)	4.6 (0.6)	6.5 (2.5)		86.6 (6.6)	120.8 (50.9)	
**BMI**			NA			<0.001
Mean (SD)	NA	NA		27.4(6.4)	31.0(7.8)	
**Gender, n (%)**			<0.001			0.012
Female	2,467 (61.0)	5,908 (37.1)		359 (52.4)	212 (44.9)	
Male	1,578 (39.0)	10,035 (62.9)		326 (47.6)	261 (55.1)	
**Age, n (%)**			<0.001			<0.001
<60	3,522(87.1)	5,985(37.5)		522(76.2)	135(28.6)	
≥60	523(12.9)	9,958(62.5)		163(23.8)	337(71.4)	
**Recent alcohol use, n (%)**			NA			<0.001
Yes	NA	NA		73 (11.8)	88 (21.7)	
No	NA	NA		544 (88.2)	318 (78.3)	
**Diabetes, n (%)**			NA			<0.001
Yes	NA	NA		28 (4.1)	167 (35.4)	
No	NA	NA		657 (95.9)	305 (64.6)	
**Hypertension, n (%)**			NA			<0.001
Yes	NA	NA		155 (22.6)	325 (68.9)	
No	NA	NA		530 (77.4)	147 (31.1)	

AST, Aspartate aminotransferase; ALT, Alanine aminotransferase; GGT, Gamma-glutamyl transferase; ALP, Alkaline phosphatase; ChE, Cholinesterase; TP, Total protein; ALB, Albumin; GLO, Globulin; A/G, Albumin/Globulin ratio; TBIL, Total bilirubin; DBIL, Direct bilirubin; IBIL, Indirect bilirubin; TBA, Total biliary acid; TC, Total cholesterol; TG, Triglyceride; HDL-C, High-density lipoprotein cholesterol; GLU, Glucose; BMI, Body Mass Index; Var, variable; NA, indicates that data for this indicator are indeed available in the database.

In cohort 2, the ASCVD group also demonstrated significantly higher mean levels of several biochemical indicators compared to the control group, including ALT (19.9 ± 13.0), ALP (90.0 ± 36.9), AST(21.6 ± 12.3), GGT (38.0 ± 50.9), TBIL (0.5 ± 0.3), GLO (3.1 ± 0.5), TG (129.3 ± 61.3), and GLU (120.8 ± 50.9) (**Table 1**). Furthermore, the ASCVD group had lower levels of ALB, A/G, and HDL-C compared to the control group (**Figure 2B**). However, total cholesterol (TC) did not exhibit significant differences between the two groups in cohort 2.

### 3.3 Correlation analysis between liver function indices and ASCVD

**Figure 3:**
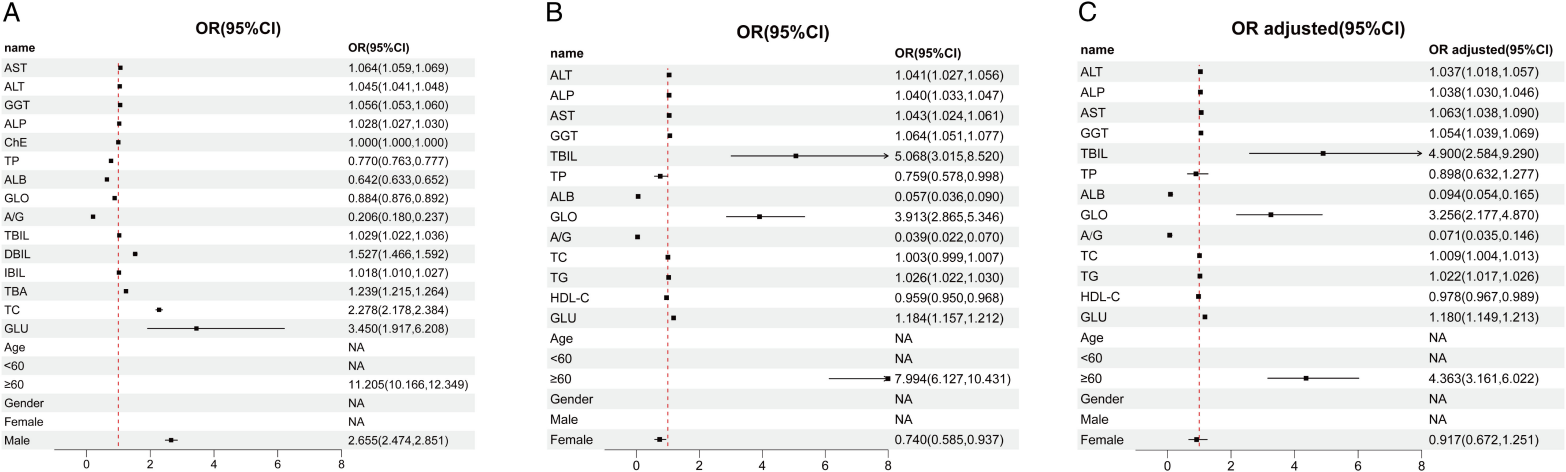
Correlation between multiple liver function indices and ASCVD. **(A)** Forest plot of univariate regression analysis for various indices in the control and ASCVD groups in cohort 1. **(B)** Forest plot of univariate regression analysis for various indices in the control and ASCVD groups in cohort 2. **(C)** Forest plot of univariate regression analysis for various indices in the control and ASCVD groups in cohort 2, adjusted for BMI, recent alcohol use, diabetes and hypertension. In this figure, the points and the length of the lines represent the odds ratios (ORs) and their 95% confidence intervals (CIs), respectively. The arrows indicate values that extend beyond the range shown on the horizontal axis below the plot. The dashed red line signifies no association between the variables (OR=1). An OR less than 1 suggests a protective effect, whereas an OR greater than 1 implies a risk factor.

To explore the potential correlation between various liver function indicators and ASCVD, we utilized raw data from both the control and ASCVD groups within the two cohorts to construct binary logistic regression models for univariate analysis (**Table 2**). The results demonstrated that in cohort 1, TP served as a protective factor, whereas DBIL, IBIL, TBA, and TC were identified as risk factors (**Figure 3A**). In cohort 2, HDL-C indicated a protective role, while TG was found to be a risk factor (**Figures 3B, C**). A notable discrepancy was observed in the GLO indicator between the two cohorts, with it exhibiting a protective effect in cohort 1 (**Figure 3A**) but a risk factor in cohort 2 (**Figures 3B, C**). Furthermore, several liver function indicators were found to potentially influence the incidence of ASCVD. In both cohorts, ALB and A/G exhibited protective effects (OR < 1), while AST, ALT, GGT, ALP, TBIL and GLU were identified as risk factors (OR > 1) (**Figure 3**). Additionally, ChE in cohort 1 (**Figure 3A**) and TP in the adjusted cohort 2 (**Figure 3C**) showed no significant correlation with ASCVD (OR = 1). However, TC in cohort 2 (**Figure 3C**), after adjusting for covariates, emerged as a risk factor for ASCVD.

**Table 2:** Univariate regression analysis of participants in two cohorts.

Var	Cohort 1				Cohort 2							
	N	OR	95%CI	P	N	OR	95%CI	P	N’	OR’	95%CI’	P’
AST	19988	1.064	[1.059,1.069]	0.000	1153	1.043	[1.024,1.061]	0.000	1001	1.063	[1.038,1.090]	0.000
ALT	19988	1.045	[1.041,1.048]	0.000	1157	1.041	[1.027,1.056]	0.000	1003	1.037	[1.018,1.057]	0.000
GGT	19987	1.056	[1.053,1.060]	0.000	1157	1.064	[1.051,1.077]	0.000	1003.0	1.054	[1.039,1.069]	0.000
ALP	19987	1.028	[1.027,1.030]	0.000	1157	1.040	[1.033,1.047]	0.000	1003	1.038	[1.030,1.046]	0.000
ChE	17604	1.000	[1.000,1.000]	0.000	NA	NA	NA	NA	NA	NA	NA	NA
TP	19988	0.770	[0.763,0.777]	0.000	1157	0.759	[0.578,0.998]	0.049	1003	0.898	[0.632,1.277]	0.549
ALB	19988	0.642	[0.633,0.652]	0.000	1157	0.057	[0.036,0.090]	0.000	1003	0.094	[0.054,0.165]	0.000
GLO	19988	0.884	[0.876,0.892]	0.000	1157	3.913	[2.865,5.346]	0.000	1003	3.256	[2.177,4.870]	0.000
A/G	19988	0.206	[0.180,0.237]	0.000	1157	0.039	[0.022,0.070]	0.000	1003	0.071	[0.035,0.146]	0.000
TBIL	19331	1.029	[1.022,1.036]	0.000	1157	5.068	[3.015,8.520]	0.000	1003	4.900	[2.584,9.290]	0.000
DBIL	19331	1.527	[1.466,1.592]	0.000	NA	NA	NA	NA	NA	NA	NA	NA
IBIL	19331	1.018	[1.010,1.027]	0.000	NA	NA	NA	NA	NA	NA	NA	NA
TBA	19330	1.239	[1.215,1.264]	0.000	NA	NA	NA	NA	NA	NA	NA	NA
TC	18318	2.278	[2.178,2.384]	0.000	1157	1.003	[0.999,1.007]	0.126	1003	1.009	[1.004,1.013]	0.001
TG	NA	NA	NA	0.000	1157	1.026	[1.022,1.030]	0.000	1003	1.022	[1.017,1.026]	0.000
HDL-C	NA		NA	NA	1158	0.959	[0.950,0.968]	0.000	1003	0.978	[0.967,0.989]	0.000
GLU	14686	3.450	[1.917,6.208]	0.000	1157	1.184	[1.157,1.212]	0.000	1003.0	1.180	[1.149,1.213]	0.000
Gender												
Female	8375			0.000	571	0.740	[0.585,0.937]	0.012	469	0.917	[0.672,1.251]	0.584
Male	11613	2.655	[2.474,2.851]	0.000	586				534			
Age												
<60	9507				657				579			
≥60	10481	11.2055	[10.166,12.349]	0.000	500	7.994	[6.127,10.431]	0.000	424	4.363	[3.161,6.022]	0.000

AST, Aspartate aminotransferase; ALT, Alanine aminotransferase; GGT, Gamma-glutamyl transferase; ALP, Alkaline phosphatase; ChE, Cholinesterase; TP, Total protein; ALB, Albumin; GLO, Globulin; A/G, Albumin/Globulin ratio; TBIL, Total bilirubin; DBIL, Direct bilirubin; IBIL, Indirect bilirubin; TBA, Total biliary acid; TC, Total cholesterol; TG, Triglyceride; HDL-C, High-density lipoprotein cholesterol; GLU, Glucose; NA, indicates that data for this indicator are indeed available in the database; Var, variable; OR, odds ratio; 95% CI, 95% confidence interval; In Cohort 2, the N’ group adjusted for ASCVD prevalence based on BMI, recent alcohol use, diabetes, hypertension; OR’, odds ratio-adjusted; 95% CI’, 95% confidence interval-adjusted.

## 4 Subgroup analysis of the association between liver function indices and ASCVD in cohort 1

Following our prior analysis, we identified correlations between several liver function indices and ASCVD in both cohorts. To delve deeper into the presence of these correlations in specific subsets or subpopulations within Cohort 1, we conducted stratified regression analyses, grouping participants in Cohort 1 into diverse subgroups according to age (<60, ≥60 years) and gender (male, female).

**Figure 4:**
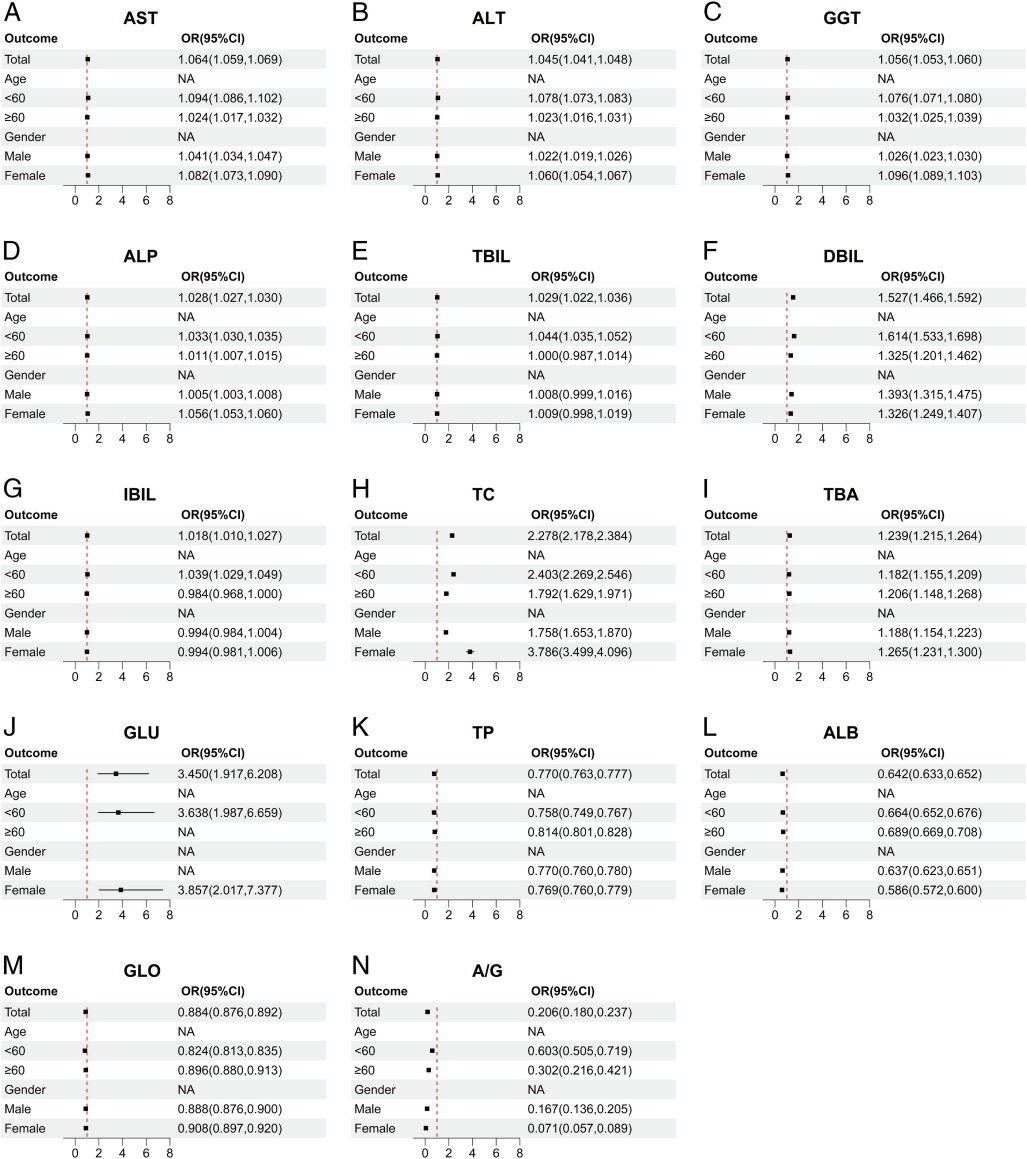
Correlation between multiple liver function indicators and ASCVD in different subgroups. **(A)** AST, Aspartate aminotransferase. **(B)** ALT, Alanine aminotransferase. **(C)** GGT, Gamma-glutamyl transferase. **(D)** ALP, Alkaline phosphatase. **(E)** TBIL, Total bilirubin. **(F)** DBIL, Direct bilirubin. **(G)** IBIL, Indirect bilirubin. **(H)** TC, Total cholesterol. **(I)** TBA, Total biliary acid. **(J)** GLU, Glucose. **(K)** TP, Total protein. **(L)** ALB, Albumin. **(M)** GLO, Globulin. **(N)** A/G, Albumin/Globulin ratio. The above figure depicts a forest plot of subgroup-stratified regression analysis in cohort 1. The subgroups are stratified by age (<60, ≥60) and gender (male, female), illustrating the OR values for various indicators across these subgroups. The points and line segments in the figure represent the OR values and their corresponding 95% confidence intervals. Arrows indicate values that extend beyond the range displayed on the horizontal axis below the figure. The red dashed line signifies no correlation between the two variables (OR=1), with OR<1 indicating a protective factor and OR>1 indicating a risk factor.

The data from Cohort 1 indicate that AST (**Figure 4A**), ALT (**Figure 4B**), GGT (**Figure 4C**), ALP (**Figure 4D**), TBIL (**Figure 4E**), DBIL (**Figure 4F**), IBIL (**Figure 4G**), and TC (**Figure 4H**) are potential risk factors for ASCVD. Notably, these indicators exhibit a higher correlation with ASCVD in individuals under 60 years old. Additionally, TBA (**Figure 4I**) and GLU (**Figure 4J**) also emerge as potential risk factors, albeit with age-stratified results that are diametrically opposed to those of the aforementioned indicators, particularly in that GLU (**Figure 4J**) loses statistical significance in individuals aged 60 and above. When subgroups are stratified by gender, AST (**Figure 4A**), ALT (**Figure 4B**), GGT (**Figure 4C**), ALP (**Figure 4D**), TBIL (**Figure 4E**), and TC (**Figure 4H**) demonstrate slightly higher OR values in females compared to males. However, for DBIL (**Figure 4F**), the OR value in females is slightly lower than in males. The remaining indicators, including TP (**Figure 4K**), ALB (**Figure 4L**), GLO (**Figure 4M**), and A/G (**Figure 4N**), consistently exhibit a protective effect across different genders and age groups.

## 5 Subgroup analysis of the association between liver function indices and ASCVD in cohort 2

To further validate the correlation between liver function indicators and ASCVD across different populations or subgroups within cohort 2, we conducted stratified regression analyses by categorizing each indicator into various subgroups based on age (<60, ≥60) and gender (male, female).

**Figure 5:**
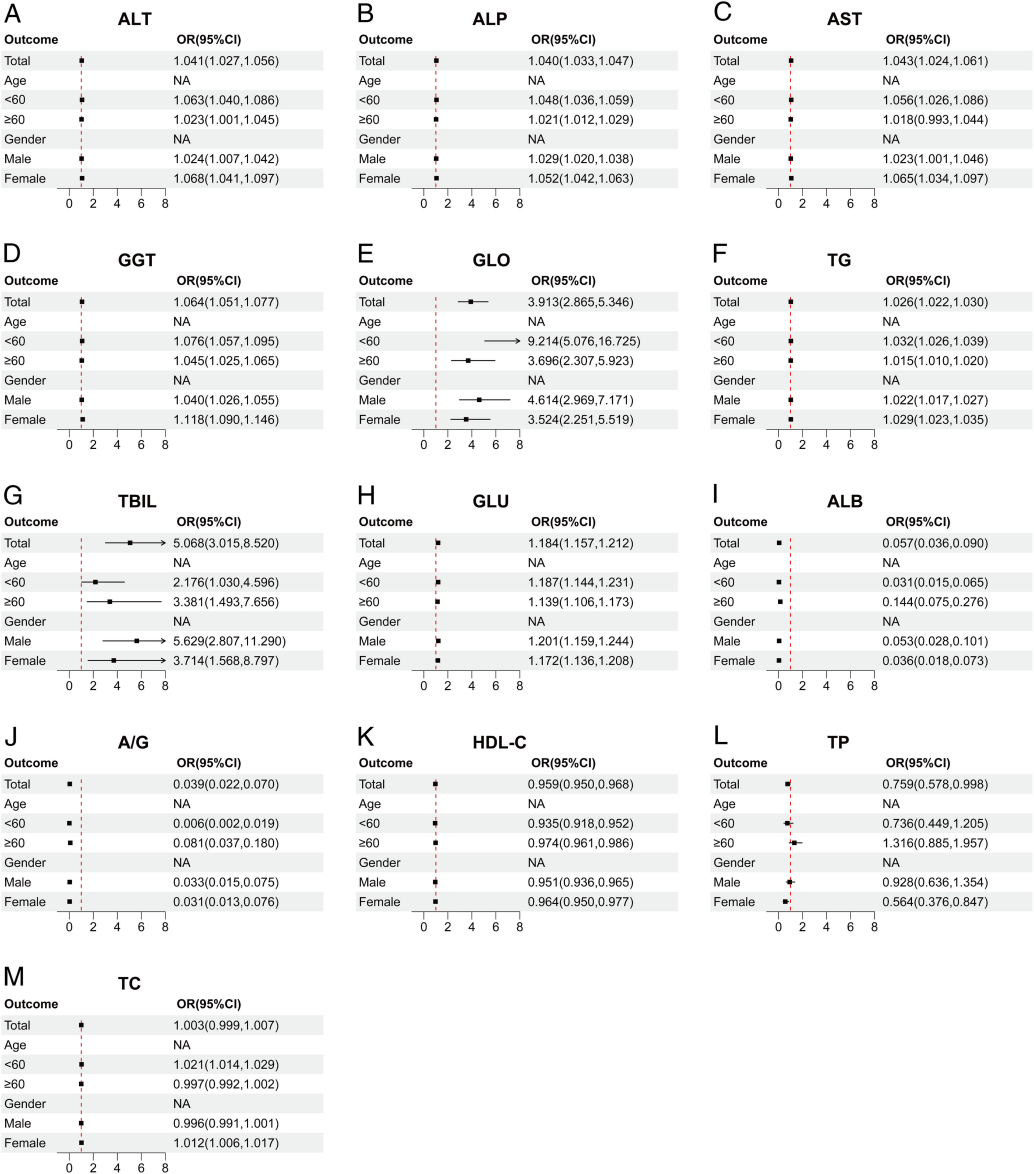
Correlation between multiple liver function indicators and ASCVD in different subgroups. **(A)** ALT, Alanine aminotransferase. **(B)** ALP, Alkaline phosphatase. **(C)** AST, Aspartate aminotransferase. **(D)** GGT, Gamma-glutamyl transferase. **(E)** GLO, Globulin. **(F)** TG, Triglyceride. **(G)** TBIL, Total bilirubin **(H)** GLU, Glucose. **(I)** ALB, Albumin. **(J)** A/G, Albumin/Globulin ratio. **(K)** HDLC, High-density lipoprotein cholesterol. **(L)** TP, Total protein. **(M)** TC, Total cholesterol. The above figure presents a forest plot of subgroup-stratified regression analysis in cohort 2. The subgroups are stratified by age (<60, ≥60) and gender (male, female), showing the OR values between different indicators and different subgroups. The points and line segments in the figure indicate the OR values and their 95% confidence intervals, respectively. Arrows indicate values that extend beyond the displayed range of the horizontal axis within the figure. The red dashed line represents no correlation between the two (OR=1); OR<1 indicates a protective factor, and OR>1 indicates a risk factor.

**Figure 6:**
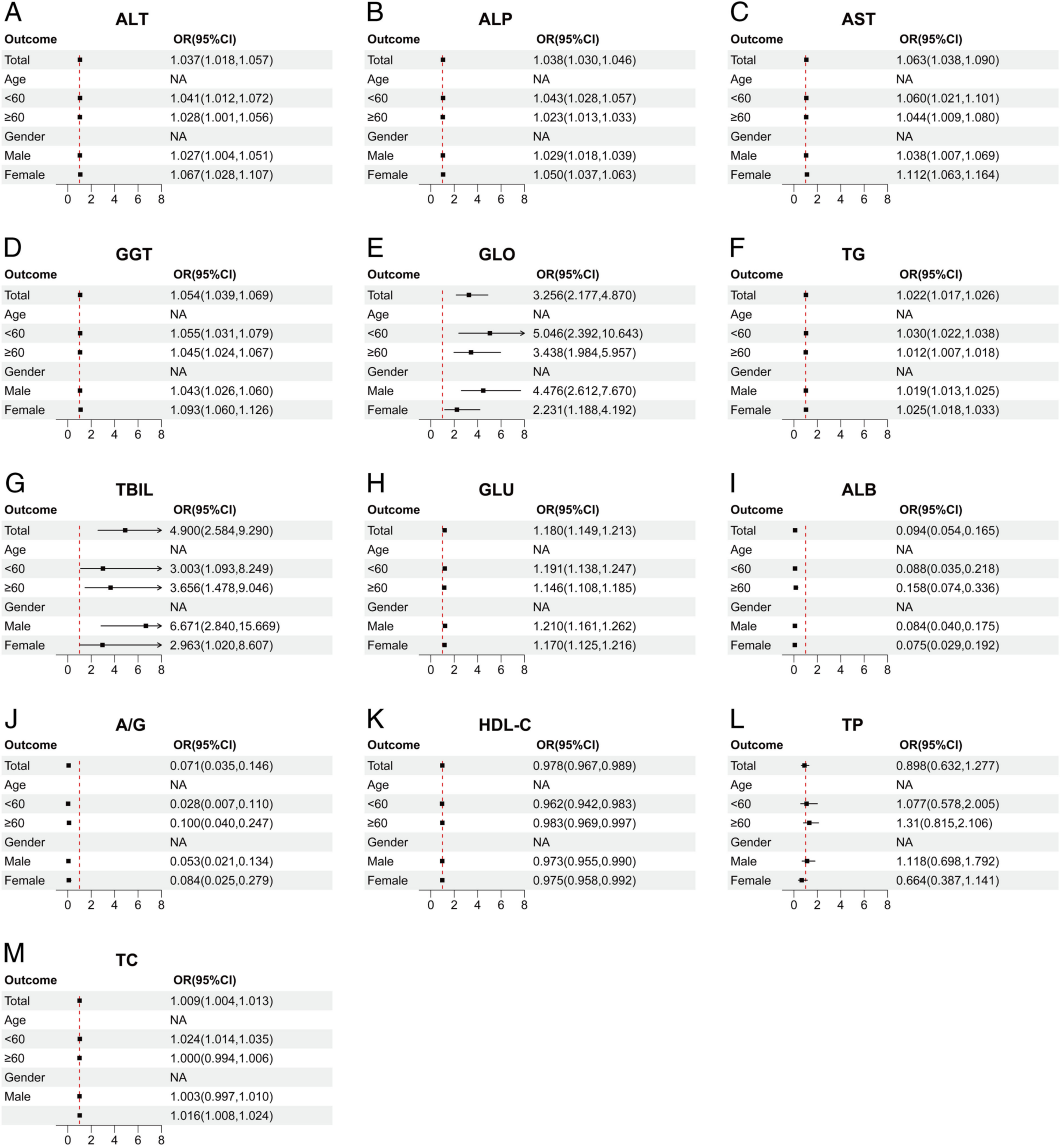
Correlations between multiple liver function indicators and ASCVD in different subgroups, adjusted for BMI, recent alcohol use, diabetes and hypertension. **(A)** ALT, Alanine aminotransferase. **(B)** ALP, Alkaline phosphatase. **(C)** AST, Aspartate aminotransferase. **(D)** GGT, Gamma-glutamyl transferase. **(E)** GLO, Globulin. **(F)** TG, Triglyceride. **(G)** TBIL, Total bilirubin **(H)** GLU, Glucose. **(I)** ALB, Albumin. **(J)** A/G, Albumin/Globulin ratio. **(K)** HDLC, High-density lipoprotein cholesterol. **(L)** TP, Total protein. **(M)** TC, Total cholesterol. The above figure presents a forest plot of subgroup-stratified regression analysis in cohort 2. The subgroups are stratified by age (<60, ≥60) and gender (male, female), showing the OR values between different indicators and different subgroups. The points and line segments in the figure indicate the OR values and their 95% confidence intervals, respectively. Arrows indicate values that extend beyond the displayed range of the horizontal axis within the figure. The red dashed line represents no correlation between the two (OR=1); OR<1 indicates a protective factor, and OR>1 indicates a risk factor.

The results of cohort 2 indicate that ALT (**Figures 5A**, **6A**), ALP (**Figures 5B**, **6B**), AST (**Figures 5C**, **6C**), GGT (**Figures 5D**, **6D**), GLO (**Figures 5E**, **6E**), and TG (**Figures 5F**, **6F**) are potential risk factors for ASCVD, regardless of covariate adjustment, and these indicators demonstrate a higher correlation with ASCVD in age strata below 60 years. Furthermore, within gender-specific subgroups, the odds ratios (ORs) for ALT (**Figures 5A**, **6A**), ALP (**Figures 5B**, **6B**), AST (**Figures 5C**, **6C**), GGT (**Figures 5D**, **6D**), and TG (**Figures 5F**, **6F**) are higher in females compared to males. Notably, TBIL (**Figures 5G**, **6G**) exhibits a significantly larger OR value in the public database than other indicators, suggesting a stronger association with ASCVD. GLU (**Figures 5H**, **6H**) also persists as a potential risk factor, but it demonstrates a higher risk in male populations and elderly individuals aged 60 years and above. The remaining indicators, namely ALB (**Figures 5I**, **6I**), A/G (**Figures 5J**, **6J**), and HDL-C (**Figures 5K**, **6K**), consistently act as protective factors across various age and gender subgroups. Additionally, TP (**Figures 5L**, **6L**) loses statistical significance across all subgroups after adjusting for covariates, while TC (**Figures 5M**, **6M**) demonstrates overall statistical significance in correlation after covariate adjustment and acts as a risk factor in female populations and age strata below 60 years.

The liver function indicators that correlated with ASCVD in the overall data for both cohorts also demonstrated varying degrees of correlation across different subgroups. Among the common liver function indicators shared by both cohorts, AST (**Figures 4A**, **5C**), ALT (**Figures 4B**, **5A**), GGT (**Figures 4C**, **5D**), ALP (**Figures 4D**, **5B**), and GLU (**Figures 4J**, **5H**) consistently emerged as risk factors across various age groups, particularly posing a higher risk in females. Furthermore, ALB (**Figures 4L**, **5I**) and A/G (**Figures 4N**, **5J**) consistently exhibited protective effects in different subgroups. However, a notable difference between the two cohorts was observed in the case of GLO (**Figures 4M**, **5E**), which played a protective role against ASCVD in cohort 1 but had an adverse effect in cohort 2.

Concurrently, in our subgroup sensitivity analysis, we utilized the E-value to report the robustness of various indicator models. Among them, the E-values for liver injury assessment indicators (ALT, ALP, AST, GGT) were relatively small (**Supplementary Table 10**), suggesting that potential confounding factors could lead to instability in the results. In contrast, liver metabolism indicators (TBIL, DBIL, GLU, TBA) exhibited larger E-values, indicating better robustness. Furthermore, the majority of protective factors had larger E-values (**Supplementary Table 10**), indicating that their protective significance is highly reliable. Therefore, based on the current results, liver function indicators demonstrate a certain correlation with ASCVD, but some indicators may be susceptible to other confounding factors. It is imperative to incorporate more ASCVD-related confounding factors into future studies for comprehensive investigation.

## 6 Discussion

Metabolic disturbances constitute a significant feature of ASCVD, and the liver, as the central organ regulating substance metabolism in the human body, plays a crucial role in the pathogenesis and progression of ASCVD. Given this, liver function assessment indicators have garnered considerable attention as potential early diagnostic tools for ASCVD, leading to numerous research achievements. However, despite studies offering some clues regarding the association between liver function and ASCVD, discrepancies persist among research findings. These discrepancies may be influenced by factors such as small sample sizes, inadequate follow-up periods, or differences in age, gender, and ethnicity. Therefore, further large-scale data validation is necessary to elucidate these connections. In this study, we employed a broader and more diversified research strategy, not only by expanding the sample size but also by integrating data from diverse ethnic backgrounds. This approach furnished more solid and reliable clinical evidence for the potential link between liver function and ASCVD, while also offering new perspectives and insights into the pathogenesis of ASCVD.

Assessment indicators of hepatocyte injury primarily encompass AST, ALT, GGT, and ALP. Clinical studies have demonstrated that these liver enzymes are significantly associated with various high-risk factors for ASCVD, such as lipid metabolism disorders and diabetes. Existing research has confirmed that these liver enzymes are linked to lipid metabolism disturbances and lipid deposition, and can predict major adverse liver outcomes in patients with metabolic dysfunction-associated fatty liver disease (MAFLD) (19). Additionally, ALT is correlated with the occurrence of diabetes (20). A higher ALT/AST ratio is independently associated with a significant increase in the risk of non-alcoholic fatty liver disease (NAFLD) and liver fibrosis (21). Thus, elevated liver enzyme levels are linked to ASCVD incidence. However, other studies have found that the association between liver enzyme levels and ASCVD is controversial, potentially influenced by genetic, environmental, and other factors. A recent meta-analysis has provided new insights into the relationship between liver enzyme levels and ASCVD across different ethnic populations. It demonstrated that the correlation between ALT and ASCVD is positive among Asian populations, but negative among North American and European populations, yielding varied outcomes for different ASCVD endpoints. Specifically, ALT is negatively correlated with coronary heart disease but positively correlated with stroke (22). In our study, results from two cohorts showed that ALT, AST, ALP, and GGT were risk factors for ASCVD in both cohorts. The discrepancy may arise due to temporal effects. The data cited in Kunutsor’s study utilized baseline liver enzyme levels from healthy populations to monitor ASCVD incidence over a subsequent period. However, these data may not account for individual variations in liver enzyme levels over time, potentially leading to an underestimation of the correlation between them (22). In contrast, our study provides robust evidence for a significant correlation between elevated liver enzyme levels and ASCVD by comparing liver enzyme levels and ASCVD incidence at the same time point in healthy individuals and ASCVD patients at baseline. In other Asian countries, such as Bangladesh and South Korea, multiple reports have also shown a significant correlation between elevated liver enzyme levels and ASCVD (2, 3). Furthermore, two studies have found that ideal cardiovascular health indicators are significantly associated with lower levels of ALT and GGT in healthy adolescents in Europe and in healthy populations in South American countries, suggesting that elevated levels of ALT and GGT may serve as risk factors for ASCVD (23, 24). However, the potential association between liver enzyme levels and ASCVD is influenced by various factors. For instance, studies have demonstrated that genetic factors may account for individual variations in plasma concentrations of liver enzymes among non-diseased and unrelated populations (25). Additionally, in individuals at risk of metabolic disorders, genetic factors continue to impact liver enzyme levels and interact with environmental factors, such as depression (26). Furthermore, the overall nutritional status of patients can also affect liver enzyme levels (27). Therefore, future studies should take into account more potential influencing factors to more precisely elucidate the true relationship between liver enzyme levels and ASCVD risk.

In the assessment of liver synthetic function indicators (ALB, GLO, A/G), studies have demonstrated that an ALB level of ≥3.75g/dL can reduce mortality among ASCVD patients, potentially aiding in risk prediction for elderly patients with stable ASCVD (7). In our study, the average ALB levels were consistently above 3.75g/dL, and ALB emerged as a protective factor for ASCVD across all age groups, further corroborating the aforementioned viewpoint. Regarding the relationship between GLO and ASCVD, a study of peritoneal dialysis patients in Taiwan, China, found that the ASCVD mortality rate was significantly higher in the high GLO group compared to the low GLO group, confirming GLO as an independent risk factor for ASCVD (8). However, in our study, the relationship between GLO and ASCVD was inconsistent across the two study cohorts. In cohort 1, GLO served as a protective factor, whereas in cohort 2, it appeared as a risk factor contributing to ASCVD occurrence. The discrepancies may stem from differences in globulin metabolism among different ethnicities. A recent study has revealed adaptive evolutionary differences in immunoglobulin heavy chain constant region genes among diverse populations, which may indirectly shed light on racial differences in globulin metabolism (28). Future research is needed to delve deeper into the potential mechanisms underlying the influence of GLO on the occurrence and development of ASCVD in various ethnic groups, as well as the specific reasons for these differences.

For the assessment of liver metabolic function, with particular focus on TBIL, numerous studies have demonstrated an independent inverse correlation between bilirubin levels and ASCVD risk (9, 10). Relevant research suggests that bilirubin deficiency may induce inflammation and impair the stability of atherosclerotic plaques (29). Additionally, bilirubin possesses antioxidant and anti-inflammatory properties, which, to some extent, slow down the development of atherosclerosis (30). However, in both cohorts, bilirubin emerged as a risk factor for ASCVD. The reasons for this discrepancy may lie in the dosage effect of bilirubin. At normal levels, bilirubin is not associated with ASCVD risk and may even reduce it (30). Conversely, abnormally elevated bilirubin levels may exert adverse effects on ASCVD (31). A large meta-analysis also revealed a U-shaped dose-response relationship between bilirubin and ASCVD, particularly among males (32). Furthermore, studies have confirmed that high levels of bilirubin can cause cytotoxicity and tissue damage, providing further evidence to explain the observed discrepancies (31). Given the unique biological mechanisms of bilirubin in the development of ASCVD, future in-depth exploration of the specific associations and mechanisms between bilirubin and ASCVD holds dual potential for both early clinical diagnosis and therapeutic strategies.

Furthermore, the relationship between traditional lipid indices (TC, HDL-C) and ASCVD risk has been widely acknowledged. Studies have indicated a positive correlation between serum TC and LDL-C levels and ASCVD mortality, as well as a negative correlation between HDL-C levels and ASCVD mortality (33). However, in the raw data of cohort two, there was no significant correlation between TC and ASCVD. Upon examining the data, we observed that the mean values of TC in both the control group and ASCVD patients fell within the normal range. Additionally, we lacked data on low-density lipoprotein cholesterol (LDL-C), which is a crucial lipoprotein subclass. Therefore, a single TC level may not be sufficient for predicting ASCVD. Notably, a series of longitudinal observational studies have offered us a fresh perspective. Researchers collected blood lipid index data (TC, LDL-C, ApoB, TC/HDL-C) from the same cohort at various time points and discovered that the degree of variation in these data was independently linked to the progression of ASCVD and adverse cardiovascular outcomes (34, 35). Another study, which focused exclusively on the correlation between TC and ASCVD, also emphasized that higher quartile TC variability was more significantly associated with ASCVD, irrespective of average TC levels or the use of lipid-lowering medications (36). Furthermore, relevant studies have pointed out that higher variability in TC levels may lead to repeated crystallization and dissolution of cholesterol crystals within coronary artery plaques (37). During the process of cholesterol crystal formation in coronary artery plaques, the enlargement of the necrotic core may lead to plaque disruption or rupture, thereby triggering further inflammatory responses (38). In our study, we investigated the association between a single measurement of TC and ASCVD. Although statistically significant differences were observed in cohort one and the adjusted cohort two, no significant correlation was found in the raw data of cohort two. This suggests that the correlation between TC and ASCVD is unstable, and blood lipid levels at a single time point may not be ideal for predicting and diagnosing ASCVD. In future research, long-term monitoring of lipid index variability will offer a more precise and dependable foundation for assessing the relationship between lipid levels and ASCVD risk.

Lastly, among other common biochemical indicators, the GLU level is recognized as an independent risk factor for ASCVD (39). Furthermore, in our study, GLU also emerged as a risk factor for ASCVD, thereby validating this perspective. In recent years, an increasing number of researchers have focused on composite indices derived from multiple indicators, which seem to provide a more comprehensive and accurate basis for early disease prediction and diagnosis. For example, in the U.S. population, a study indicated a U-shaped association between the baseline triglyceride-glucose index (TyG) and all-cause mortality in patients with CVD. The TyG index is a predictor of cardiovascular and all-cause mortality in patients with diabetes or prediabetes who have ASCVD (40). The development of more comprehensive and diverse composite indices for early disease prevention and diagnosis in the future could thereby potentially offer new viable treatment options for clinical practice, improving patients’ survival status and quality of life.

In recent years, the significance of liver assessment indicators in the field of ASCVD has become increasingly prominent, providing new insights into our understanding of the pathogenesis of ASCVD. Among them, liver enzyme indicators (AST, ALT, GGT, ALP) are closely associated with lipid metabolism disorders, a high-risk factor for ASCVD, which may represent one of the underlying mechanisms through which liver dysfunction promotes the onset and progression of ASCVD (19). Meanwhile, ALB as an important liver-synthesized protein, should not be overlooked for its protective role at normal levels. The anti-inflammatory and antioxidant properties of ALB contribute to slowing the development of atherosclerosis, thereby reducing the risk of ASCVD to a certain extent (41). It is noteworthy that when bilirubin levels rise abnormally, its original anti-inflammatory and antioxidant characteristics can transform into cytotoxicity and tissue damage, emerging as a new risk factor for ASCVD (31). In addition, traditional lipid indicators (TC, LDL-C, HDL-C) also play a crucial role in the pathogenesis of ASCVD. These lipid indicators, induced by adverse factors, are prone to depositing on the vessel wall, triggering local inflammatory responses and oxidative stress, which facilitate the formation of atherosclerotic plaques and subsequently increase the risk of ASCVD (42). In summary, liver assessment indicators are indispensable in advancing our understanding of the pathogenesis of ASCVD and serving as potential diagnostic or prognostic biomarkers. By comprehensively evaluating these indicators, we can gain a more comprehensive understanding of a patient’s metabolic status, providing more precise guidance for the prevention, diagnosis, and treatment of ASCVD.

Despite these findings, our study still has certain limitations that need to be addressed. Firstly, due to practical and ethical constraints, Cohort 1 was unable to collect covariate data, potentially leading to an inability to control for certain unaccounted confounding factors. This makes it difficult to ascertain the contribution of the aforementioned indicators to disease prevention and early diagnosis, especially in the presence of other more significant risk factors. To mitigate this issue, we included as many ASCVD-relevant covariates as possible in Cohort 2 for adjustment, aiming to better validate and supplement our findings. Secondly, although our study results indicate statistically significant correlations between the relevant biochemical indicators and ASCVD, the OR values of some indicators are close to 1, implying that their association with ASCVD may not be substantial and their practical diagnostic guidance may be limited. Furthermore, the causal relationships between various indicators and ASCVD over time remain unclear. A deeper understanding of the exact impacts and roles of each indicator in the onset and progression of the disease, as well as clarifying the causal relationships among them, will facilitate the development of a more comprehensive multi-factor risk assessment and prediction model in the future, offering new avenues for subsequent research.

Despite the existing controversies among various research findings, the relationship between liver function assessment indicators and ASCVD remains worth exploring. In our study, by expanding the sample size, stratifying by age and gender, and investigating differences among different racial groups, we aimed to resolve the controversies and were able to obtain more authentic and reliable results. In summary, our study results indicate differences in liver function assessment indicators between ASCVD patients and healthy volunteers. In the future, we will attempt to conduct a more in-depth analysis of the correlation and causation between the two by conducting long-term follow-up studies, incorporating the time effect, and collecting clinical endpoint information from participants to carry out more rigorous and reliable prospective studies on the predictive diagnosis of ASCVD using liver function indicators. Meanwhile, to jointly explore and investigate their correlation with ASCVD, it is necessary to incorporate more relevant indicators. For example, collecting additional lifestyle and complex metabolic indicators as covariates maximizes the exclusion of confounding factors. Furthermore, integrating genetic information for Mendelian Randomization (MR) analysis, and utilizing ultrasonography, magnetic resonance imaging, and corresponding liver function scores to establish multi-dimensional machine learning models, can thereby predict and diagnose ASCVD. A deeper understanding of the biological functions, mechanisms of action, and pathological relationships between various liver function indicators and ASCVD will bring new strategies and insights into the prevention and treatment of ASCVD. This further underscores the significant value of liver function assessment indicators in maintaining human health and opens up new avenues for future medical practice.

## 7 Conclusion

Our research reveals that there exist differences in multiple liver function indicators between healthy individuals and those with ASCVD. Through a large-sample cohort study, regression analysis was conducted, demonstrating correlations between several liver function indicators and ASCVD. However, some of these correlations are relatively weak and susceptible to confounding factors, suggesting that their application in predicting ASCVD associations may lack significant clinical relevance. In the future, a multi-dimensional approach encompassing various examination and testing items should be employed to meet the needs of this clinical diagnosis. By elucidating the intricate relationship between liver metabolism and cardiovascular diseases, our study offers a novel perspective for the diagnosis and prevention of ASCVD.

## Data Availability

The data analyzed in this study is subject to the following licenses/restrictions: Due to ethical constraints, data from cohort 1 in this study are available upon request from the corresponding author. Data used in cohort 2 of this study are publicly available from the Centers for Disease Control and Prevention at https://wwwn.cdc.gov/nchs/nhanes/continuousnhanes/default.aspx?Cycle=2017-2020. Requests to access these datasets should be directed to Taiyu Zhai: zhaitaiyu@jlu.edu.cn.
