# Supplementary material for: Liver function differences in atherosclerotic cardiovascular disease: a multi-ethnic dual-cohort retrospective study
Source: Front Endocrinol (Lausanne). 2025 Mar 24;16:1558872. doi: 10.3389/fendo.2025.1558872 (PMC11973100; doi:10.3389/fendo.2025.1558872)
Supplement: Supplementary file 1 [file Table1.docx]

**Liver Function Differences in Atherosclerotic Cardiovascular Disease: A multi-ethnic dual-cohort retrospective study**

Table S1. Tests and diagnostic criteria for liver function indicators

| **Liver Function Indicators** | **Range of Reference Values** | | | |
| --- | --- | --- | --- | --- |
|  | **Cohort1** | | **Cohort2** | |
|  | **Male** | **Female** | **Male** | **Female** |
| ALT | 9-50U/L | 7-40U/L | 0-40U/L | 0-31U/L |
| ALP | 45-125U/L | 35-100U/L | 40-129U/L | 35-104U/L |
| AST | 15-40U/L | 13-35U/L | 0-37U/L | 0-31U/L |
| GGT | 10-60U/L | 7-45U/L | 11-51U/L | 7-33U/L |
| ChE | 4620-11500  U/L | 3930-1080  U/L | NA | |
| TP | 65-85g/L | | 6.6-8.7g/dL | |
| ALB | 40-55g/L | | 3.5-5.0g/dL | |
| GLO | 20-40g/L | | 20-35g/L | |
| A/G | 1.2-2.4 | | NA | |
| TC | 2.6-6.0mmol/L | | 0-200mg/dL | |
| TG | 0.28-1.80mmol/L | | 0-150mg/dL | |
| TBIL | 0-26μmol/L | 0-21μmol/L | ＞1.0mg/dL | |
| DBIL | 0-6.8μmol/L | | NA | |
| IBIL | 5-20μmol/L | | NA | |
| TBA | 0-10μmol/L | | NA | |
| GLU | 3.9-6.1mmol/L | | 60-99mg/dL | |
| HDL-C | 0.76-2.10mmol/L | | ＞40mg/dL | 50mg/dL |
| LDL-C | 2.06-3.10mmol/L | | NA | |
| ApoA1 | 1.00-1.75g/L | 1.00-2.05g/L | NA | |
| ApoB | 0.6-1.1g/L | | NA | |
| Lp(a) | 0-0.3g/L | | NA | |
| HCY | 0-20μmol/L | 0-15μmol/L | NA | |

**Abbreviations: AST**: Aspartate aminotransferase. **ALT**: Alanine aminotransferase. **GGT**: Gamma-glutamyl transferase. **ALP**: Alkaline phosphatase. **ChE**: Cholinesterase. **TP**: Total protein. **ALB**: Albumin. **GLO**: Globulin. **A/G**: Albumin/Globulin ratio. **TBIL**: Total bilirubin. **DBIL**: Direct bilirubin. **IBIL**: Indirect bilirubin. **TBA**: Total biliary acid. **TC**: Total cholesterol. **TG**: Triglyceride. **HDL-C**: High-density lipoprotein cholesterol. **LDL-C**: Low-density lipoprotein cholesterol. **ApoA1**: Apolipoprotein A-I. **ApoB**: Apolipoprotein B. **Lp(a)**: Lipoprotein(a). **HCY**: Homocysteine. **GLU**: Glucose. **NA**: Not available

Table S2. Descriptive statistics for cohort 1 samples (categorical data)

| **Variable** | **Total Count** | **Categories** | **Most Frequent Category** | **Frequency** | **Missing Rate %** |
| --- | --- | --- | --- | --- | --- |
| Group | 19988 | 2 | ASCVD | 15943 | 0.000 |
| Gender | 19988 | 2 | Male | 11613 | 0.000 |
| Age | 19988 | 2 | ≥60 | 10481 | 0.000 |

Table S3. Descriptive statistics for cohort 1 samples (count data)

| **Variables** | **Total Count** | **Mean** | **Median** | **25% Percentile** | **75% Percentile** | **Standard Deviation** | **Minimum** | **Maximum** | **Missing Rate %** |
| --- | --- | --- | --- | --- | --- | --- | --- | --- | --- |
| AST | 19988 | 49.881 | 22.150 | 18.000 | 30.400 | 155.439 | 4.000 | 9033.100 | 0.000 |
| ALT | 19988 | 29.319 | 20.700 | 14.600 | 31.400 | 74.569 | 0.200 | 5831.000 | 0.000 |
| GGT | 19987 | 38.603 | 26.400 | 18.100 | 41.300 | 52.779 | 1.600 | 2621.100 | 0.005 |
| ALP | 19987 | 80.899 | 76.600 | 63.300 | 92.600 | 32.072 | 15.800 | 1367.600 | 0.005 |
| ChE | 17604 | 7882.814 | 7903.000 | 6820.750 | 9002.000 | 1738.864 | 692.000 | 27258.000 | 11.927 |
| TP | 19988 | 68.322 | 68.400 | 63.400 | 73.500 | 6.785 | 35.400 | 95.800 | 0.000 |
| ALB | 19988 | 41.001 | 41.000 | 37.700 | 44.700 | 4.879 | 16.700 | 56.600 | 0.000 |
| GLO | 19988 | 27.320 | 27.100 | 24.600 | 29.800 | 3.969 | 13.000 | 58.700 | 0.000 |
| A/G | 19988 | 1.528 | 1.520 | 1.370 | 1.680 | 0.256 | 0.420 | 3.230 | 0.000 |
| TBIL | 19331 | 14.337 | 13.100 | 10.200 | 16.900 | 6.883 | 2.600 | 272.300 | 3.287 |
| DBIL | 19331 | 2.756 | 2.400 | 1.800 | 3.100 | 2.404 | 0.200 | 149.400 | 3.287 |
| IBIL | 19331 | 11.581 | 10.600 | 8.200 | 13.700 | 5.078 | 0.000 | 127.300 | 3.287 |
| TBA | 19330 | 3.956 | 2.900 | 1.800 | 4.600 | 5.001 | 0.000 | 230.400 | 3.292 |
| TC | 18318 | 4.111 | 3.920 | 3.250 | 4.810 | 1.197 | 1.120 | 14.480 | 8.355 |
| TG | 15032 | 1.722 | 1.400 | 1.010 | 1.990 | 1.299 | 0.120 | 30.370 | h |
| HDL-C | 15032 | 1.070 | 1.040 | 0.890 | 1.210 | 0.255 | 0.310 | 2.640 | 24.795 |
| LDL-C | 15032 | 2.642 | 2.550 | 2.000 | 3.210 | 0.915 | 0.360 | 8.060 | 24.795 |
| ApoA1 | 1478 | 1.248 | 1.230 | 1.080 | 1.370 | 0.233 | 0.460 | 2.320 | 92.606 |
| ApoB | 1478 | 0.851 | 0.810 | 0.660 | 1.030 | 0.296 | 0.170 | 2.810 | 92.606 |
| Lp(a) | 1478 | 0.261 | 0.130 | 0.050 | 0.340 | 0.326 | 0.000 | 2.350 | 92.606 |
| HCY | 794 | 14.693 | 11.865 | 9.570 | 15.788 | 10.460 | 2.030 | 106.810 | 96.028 |
| GLU | 14686 | 6.462 | 5.680 | 5.030 | 6.960 | 2.518 | 1.580 | 29.790 | 26.526 |

**Abbreviations: AST**: Aspartate aminotransferase. **ALT**: Alanine aminotransferase. **GGT**: Gamma-glutamyl transferase. **ALP**: Alkaline phosphatase. **ChE**: Cholinesterase. **TP**: Total protein. **ALB**: Albumin. **GLO**: Globulin. **A/G**: Albumin/Globulin ratio. **TBIL**: Total bilirubin. **DBIL**: Direct bilirubin. **IBIL**: Indirect bilirubin. **TBA**: Total biliary acid. **TC**: Total cholesterol. **TG**: Triglyceride. **HDL-C**: High-density lipoprotein cholesterol. **LDL-C**: Low-density lipoprotein cholesterol. **ApoA1**: Apolipoprotein A-I. **ApoB**: Apolipoprotein B. **Lp(a)**: Lipoprotein(a). **HCY**: Homocysteine. **GLU**: Glucose.

Table S4. Descriptive statistics for cohort 2 samples (categorical data)

| **Variable** | **Total Count** | **Categories** | **Most Frequent Category** | **Frequency** | **Missing Rate %** |
| --- | --- | --- | --- | --- | --- |
| Gender | 1157 | 2 | Male | 586 | 0.000 |
| Group | 1157 | 2 | Control | 685 | 0.000 |
| Age | 1157 | 2 | ＜60 | 657 | 0.000 |
| Recent alcohol use | 1157 | 3 | No | 862 | 11.582 |
| Smoking status | 1157 | 4 | NA | 627 | 54.192 |
| Diabetes | 1157 | 2 | No | 962 | 0.000 |
| Hypertension | 1157 | 2 | No | 677 | 0.000 |

Table S5. Descriptive statistics for cohort 2 samples (count data)

| **Variables** | **Total Count** | **Mean** | **Median** | **25% Percentile** | **75% Percentile** | **Standard Deviation** | **Minimum** | **Maximum** | **Missing Rate %** |
| --- | --- | --- | --- | --- | --- | --- | --- | --- | --- |
| ALT | 1157 | 17.904 | 16.000 | 12.000 | 21.000 | 9.864 | 3.000 | 166.000 | 0.000 |
| ALP | 1157 | 77.064 | 72.000 | 59.000 | 88.000 | 28.893 | 16.000 | 347.000 | 0.000 |
| AST | 1153 | 20.167 | 19.000 | 16.000 | 22.000 | 8.824 | 6.000 | 188.000 | 0.346 |
| GGT | 1157 | 26.361 | 19.000 | 13.000 | 26.000 | 34.484 | 7.000 | 467.000 | 0.000 |
| TBIL | 1157 | 0.461 | 0.400 | 0.300 | 0.600 | 0.253 | 0.100 | 2.900 | 0.000 |
| TP | 1157 | 7.067 | 7.100 | 6.800 | 7.400 | 0.431 | 5.700 | 8.700 | 0.000 |
| ALB | 1157 | 4.055 | 4.100 | 3.800 | 4.300 | 0.353 | 2.400 | 5.400 | 0.000 |
| GLO | 1157 | 3.013 | 3.000 | 2.700 | 3.300 | 0.438 | 2.000 | 6.000 | 0.000 |
| A/G | 1157 | 1.379 | 1.375 | 1.219 | 1.536 | 0.258 | 0.433 | 2.571 | 0.000 |
| TC | 1157 | 168.280 | 168.000 | 148.000 | 187.000 | 31.926 | 77.000 | 438.000 | 0.000 |
| TG | 1157 | 103.262 | 93.000 | 70.000 | 125.000 | 49.846 | 30.000 | 410.000 | 0.000 |
| LDL-C | 488 | 96.121 | 90.500 | 69.000 | 120.250 | 36.904 | 7.000 | 354.000 | 57.822 |
| HDL-C | 1157 | 56.624 | 55.000 | 47.000 | 65.000 | 14.407 | 24.000 | 124.000 | 0.000 |
| GLU | 1157 | 100.541 | 91.000 | 85.000 | 99.000 | 36.963 | 49.000 | 403.000 | 0.000 |
| BMI | 1130 | 28.802 | 27.800 | 23.800 | 32.100 | 7.195 | 15.500 | 65.800 | 2.334 |

**Abbreviations: ALT**: Alanine aminotransferase. **ALP**: Alkaline phosphatase. **AST**: Aspartate aminotransferase. **GGT**: Gamma-glutamyl transferase. **TBIL**: Total bilirubin. **TP**: Total protein. **ALB**: Albumin. **GLO**: Globulin. **A/G**: Albumin/Globulin ratio. **TC**: Total cholesterol. **TG**: Triglyceride. **LDL-C**: Low-density lipoprotein cholesterol. **HDL-C**: High-density lipoprotein cholesterol. **GLU**: Glucose. **BMI**: Body Mass Index.

Table S6. Baseline analysis of cohort 1 samples

| **Variable** | **Group** | | | **p-value^b^** |
| --- | --- | --- | --- | --- |
|  | **Overall**  N = 19,988^a^ | **Control**  N = 4,045^a^ | **ASCVD**  N = 15,943^a^ |  |
| **AST** |  |  |  | <0.001 |
| Median (Q1, Q3) | 22.2 (18.0, 30.4) | 20.0 (17.2, 23.3) | 23.2 (18.3, 34.6) |  |
| Mean (SD) | 49.9 (155.4) | 20.6 (4.5) | 57.3 (173.2) |  |
| **ALT** |  |  |  | <0.001 |
| Median (Q1, Q3) | 20.7 (14.6, 31.4) | 17.3 (13.0, 23.8) | 21.9 (15.2, 34.0) |  |
| Mean (SD) | 29.3 (74.6) | 19.4 (8.5) | 31.8 (83.2) |  |
| **GGT** |  |  |  | <0.001 |
| Median (Q1, Q3) | 26.4 (18.1, 41.3) | 19.1 (14.1, 27.5) | 28.7 (19.7, 46.0) |  |
| Mean (SD) | 38.6 (52.8) | 22.1 (10.6) | 42.8 (58.1) |  |
| (Missing) | 1 | 0 | 1 |  |
| **ALP** |  |  |  | <0.001 |
| Median (Q1, Q3) | 76.6 (63.3, 92.6) | 68.8 (57.5, 80.9) | 79.1 (65.2, 95.9) |  |
| Mean (SD) | 80.9 (32.1) | 69.9 (16.4) | 83.7 (34.4) |  |
| (Missing) | 1 | 0 | 1 |  |
| **ChE** |  |  |  | <0.001 |
| Median (Q1, Q3) | 7,903.0 (6,820.8, 9,002.0) | 8,266.0 (7,319.8, 9,344.8) | 7,859.0 (6,768.0, 8,958.8) |  |
| Mean (SD) | 7,882.8 (1,738.9) | 8,328.8 (1,400.0) | 7,836.3 (1,764.0) |  |
| (Missing) | 2,384 | 2,383 | 1 |  |
| **TP** |  |  |  | <0.001 |
| Median (Q1, Q3) | 68.4 (63.4, 73.5) | 74.7 (72.4, 77.2) | 66.4 (62.2, 71.1) |  |
| Mean (SD) | 68.3 (6.8) | 74.8 (3.5) | 66.7 (6.4) |  |
| **ALB** |  |  |  | <0.001 |
| Median (Q1, Q3) | 41.0 (37.7, 44.7) | 45.9 (44.3, 47.5) | 39.7 (37.0, 42.8) |  |
| Mean (SD) | 41.0 (4.9) | 45.9 (2.4) | 39.8 (4.5) |  |
| **GLO** |  |  |  | <0.001 |
| Median (Q1, Q3) | 27.1 (24.6, 29.8) | 28.7 (26.8, 30.9) | 26.6 (24.2, 29.3) |  |
| Mean (SD) | 27.3 (4.0) | 28.9 (3.0) | 26.9 (4.1) |  |
| **A/G** |  |  |  | <0.001 |
| Median (Q1, Q3) | 1.5 (1.4, 1.7) | 1.6 (1.5, 1.7) | 1.5 (1.3, 1.7) |  |
| Mean (SD) | 1.5 (0.3) | 1.6 (0.2) | 1.5 (0.3) |  |
| **TBIL** |  |  |  | 0.12 |
| Median (Q1, Q3) | 13.1 (10.2, 16.9) | 13.1 (10.5, 16.1) | 13.1 (10.1, 17.0) |  |
| Mean (SD) | 14.3 (6.9) | 13.5 (3.9) | 14.5 (7.4) |  |
| (Missing) | 657 | 657 | 0 |  |
| **DBIL** |  |  |  | <0.001 |
| Median (Q1, Q3) | 2.4 (1.8, 3.1) | 2.2 (1.8, 2.7) | 2.5 (1.9, 3.3) |  |
| Mean (SD) | 2.8 (2.4) | 2.3 (0.7) | 2.9 (2.6) |  |
| (Missing) | 657 | 657 | 0 |  |
| **IBIL** |  |  |  | 0.007 |
| Median (Q1, Q3) | 10.6 (8.2, 13.7) | 10.9 (8.7, 13.5) | 10.5 (8.1, 13.7) |  |
| Mean (SD) | 11.6 (5.1) | 11.2 (3.3) | 11.7 (5.4) |  |
| (Missing) | 657 | 657 | 0 |  |
| **TBA** |  |  |  | <0.001 |
| Median (Q1, Q3) | 2.9 (1.8, 4.6) | 2.3 (1.6, 3.4) | 3.0 (1.9, 4.9) |  |
| Mean (SD) | 4.0 (5.0) | 2.7 (1.6) | 4.2 (5.4) |  |
| (Missing) | 658 | 657 | 1 |  |
| **TC** |  |  |  | <0.001 |
| Median (Q1, Q3) | 3.9 (3.3, 4.8) | 3.3 (3.0, 3.7) | 4.1 (3.4, 5.0) |  |
| Mean (SD) | 4.1 (1.2) | 3.4 (0.6) | 4.3 (1.2) |  |
| (Missing) | 1,670 | 759 | 911 |  |
| **GLU** |  |  |  | <0.001 |
| Median (Q1, Q3) | 5.7 (5.0, 7.0) | 4.5 (4.0, 5.0) | 5.7 (5.0, 7.0) |  |
| Mean (SD) | 6.5 (2.5) | 4.6 (0.6) | 6.5 (2.5) |  |
| (Missing) | 5,302 | 4,033 | 1,269 |  |
| **Gender, n (%)** |  |  |  | <0.001 |
| Female | 8,375 (41.9) | 2,467 (61.0) | 5,908 (37.1) |  |
| Male | 11,613 (58.1) | 1,578 (39.0) | 10,035 (62.9) |  |
| **Age, n (%)** |  |  |  | <0.001 |
| ＜60 | 9,507 (47.6) | 3,522 (87.1) | 5,985 (37.5) |  |
| ≥60 | 10,481 (52.4) | 523 (12.9) | 9,958 (62.5) |  |

**Abbreviations: AST**: Aspartate aminotransferase. **ALT**: Alanine aminotransferase. **GGT**: Gamma-glutamyl transferase. **ALP**: Alkaline phosphatase. **ChE**: Cholinesterase. **TP**: Total protein. **ALB**: Albumin. **GLO**: Globulin. **A/G**: Albumin/Globulin ratio. **TBIL**: Total bilirubin. **DBIL**: Direct bilirubin. **IBIL**: Indirect bilirubin. **TBA**: Total biliary acid. **TC**: Total cholesterol. **GLU**: Glucose.

^a^ Median (IQR) or Frequency (%).

^b^ Wilcoxon rank sum test; Pearson's Chi-squared test.

Table S7. Baseline analysis of the cohort 2 sample

| **Variable** |  | **Group** |  | **p-value^b^** |
| --- | --- | --- | --- | --- |
|  | **Overall**  N = 1,157^a^ | **Control**  N = 685^a^ | **ASCVD**  N = 472^a^ |  |
| **ALT** |  |  |  | <0.001 |
| Median (Q1, Q3) | 16.0 (12.0, 21.0) | 15.0 (12.0, 20.0) | 16.0 (13.0, 23.5) |  |
| Mean (SD) | 17.9 (9.9) | 16.5 (6.6) | 19.9 (13.0) |  |
| **ALP** |  |  |  | <0.001 |
| Median (Q1, Q3) | 72.0 (59.0, 88.0) | 66.0 (56.0, 79.0) | 83.0 (67.5, 103.0) |  |
| Mean (SD) | 77.1 (28.9) | 68.2 (16.7) | 90.0 (36.9) |  |
| **AST** |  |  |  | 0.043 |
| Median (Q1, Q3) | 19.0 (16.0, 22.0) | 18.0 (16.0, 22.0) | 19.0 (16.0, 24.0) |  |
| Mean (SD) | 20.2 (8.8) | 19.2 (5.0) | 21.6 (12.3) |  |
| (Missing) | 4 | 2 | 2 |  |
| **GGT** |  |  |  | <0.001 |
| Median (Q1, Q3) | 19.0 (13.0, 26.0) | 17.0 (12.0, 23.0) | 23.0 (16.0, 36.0) |  |
| Mean (SD) | 26.4 (34.5) | 18.4 (8.4) | 38.0 (50.9) |  |
| **TBIL** |  |  |  | <0.001 |
| Median (Q1, Q3) | 0.4 (0.3, 0.6) | 0.4 (0.3, 0.5) | 0.5 (0.3, 0.6) |  |
| Mean (SD) | 0.5 (0.3) | 0.4 (0.2) | 0.5 (0.3) |  |
| **TP** |  |  |  | 0.017 |
| Median (Q1, Q3) | 7.1 (6.8, 7.4) | 7.1 (6.8, 7.4) | 7.0 (6.7, 7.4) |  |
| Mean (SD) | 7.1 (0.4) | 7.1 (0.4) | 7.0 (0.5) |  |
| **ALB** |  |  |  | <0.001 |
| Median (Q1, Q3) | 4.1 (3.8, 4.3) | 4.2 (4.0, 4.4) | 3.9 (3.7, 4.1) |  |
| Mean (SD) | 4.1 (0.4) | 4.2 (0.3) | 3.9 (0.4) |  |
| **GLO** |  |  |  | <0.001 |
| Median (Q1, Q3) | 3.0 (2.7, 3.3) | 2.9 (2.7, 3.2) | 3.1 (2.8, 3.4) |  |
| Mean (SD) | 3.0 (0.4) | 2.9 (0.3) | 3.1 (0.5) |  |
| **A/G** |  |  |  | <0.001 |
| Median (Q1, Q3) | 1.4 (1.2, 1.5) | 1.4 (1.3, 1.6) | 1.3 (1.1, 1.4) |  |
| Mean (SD) | 1.4 (0.3) | 1.5 (0.2) | 1.3 (0.3) |  |
| **TC** |  |  |  | 0.357 |
| Median (Q1, Q3) | 168.0 (148.0, 187.0) | 170.0 (152.0, 185.0) | 164.0 (139.5, 197.0) |  |
| Mean (SD) | 168.3 (31.9) | 167.1 (21.7) | 170.0 (42.6) |  |
| **TG** |  |  |  | <0.001 |
| Median (Q1, Q3) | 93.0 (70.0, 125.0) | 82.0 (62.0, 105.0) | 118.0 (84.0, 158.0) |  |
| Mean (SD) | 103.3 (49.8) | 85.3 (28.5) | 129.3 (61.3) |  |
| **HDL-C** |  |  |  | <0.001 |
| Median (Q1, Q3) | 55.0 (47.0, 65.0) | 58.0 (51.0, 67.0) | 49.0 (41.0, 60.0) |  |
| Mean (SD) | 56.6 (14.4) | 59.7 (12.2) | 52.1 (16.1) |  |
| **GLU** |  |  |  | <0.001 |
| Median (Q1, Q3) | 91.0 (85.0, 99.0) | 87.0 (83.0, 91.0) | 103.5 (93.0, 125.0) |  |
| Mean (SD) | 100.5 (37.0) | 86.6 (6.6) | 120.8 (50.9) |  |
| **BMI** |  |  |  | <0.001 |
| Median (Q1, Q3) | 27.8 (23.8, 32.1) | 26.5 (22.8, 30.0) | 29.2 (25.8, 34.7) |  |
| Mean (SD) | 28.8 (7.2) | 27.4 (6.4) | 31.0 (7.8) |  |
| (Missing) | 27 | 7 | 20 |  |
| **Gender, n (%)** |  |  |  | 0.012 |
| Male | 586 (50.6) | 326 (47.6) | 260 (55.1) |  |
| Female | 571 (49.4) | 359 (52.4) | 212 (44.9) |  |
| **Age, n (%)** |  |  |  | <0.001 |
| ＜60 | 657 (56.8) | 522 (76.2) | 135 (28.6) |  |
| ≥60 | 500 (43.2) | 163 (23.8) | 337 (71.4) |  |
| **Recent alcohol use, n (%)** |  |  |  | <0.001 |
| Yes | 161 (15.7) | 73 (11.8) | 88 (21.7) |  |
| No | 862 (84.3) | 544 (88.2) | 318 (78.3) |  |
| (Missing) | 134 | 68 | 66 |  |
| **Diabetes, n (%)** |  |  |  | <0.001 |
| Yes | 195 (16.9) | 28 (4.1) | 167 (35.4) |  |
| No | 962 (83.1) | 657 (95.9) | 305 (64.6) |  |
| **Hypertension, n (%)** |  |  |  | <0.001 |
| Yes | 480 (41.5) | 155 (22.6) | 325 (68.9) |  |
| No | 677 (58.5) | 530 (77.4) | 147 (31.1) |  |

**Abbreviations: ALT**: Alanine aminotransferase. **ALP**: Alkaline Phosphatase. **AST**: Aspartate aminotransferase**. GGT**: Gamma-glutamyl transferase. **TBIL**: Total bilirubin. **TP**: Total protein. **ALB**: Albumin. **GLO**: Globulin. **A/G**: Albumin/Globulin ratio. **TC**: Total cholesterol. **TG**: Triglyceride. **HDL-C**: High-density lipoprotein cholesterol. **GLU**: Glucose. **BMI**: Body Mass Index.

^a^ Median (IQR) or Frequency (%).

^b^ Wilcoxon rank sum test; Pearson's Chi-squared test.

Table S8. One-way regression analysis of cohort 1 samples

| **name** | **N** | **OR** | **95%CI** | **P-value** |
| --- | --- | --- | --- | --- |
| AST | 19988 | 1.064 | [1.059,1.069] | 0.000 |
| ALT | 19988 | 1.045 | [1.041,1.048] | 0.000 |
| GGT | 19987 | 1.056 | [1.053,1.060] | 0.000 |
| ALP | 19987 | 1.028 | [1.027,1.030] | 0.000 |
| ChE | 17604 | 1.000 | [1.000,1.000] | 0.000 |
| TP | 19988 | 0.770 | [0.763,0.777] | 0.000 |
| ALB | 19988 | 0.642 | [0.633,0.652] | 0.000 |
| GLO | 19988 | 0.884 | [0.876,0.892] | 0.000 |
| A/G | 19988 | 0.206 | [0.180,0.237] | 0.000 |
| TBIL | 19331 | 1.029 | [1.022,1.036] | 0.000 |
| DBIL | 19331 | 1.527 | [1.466,1.592] | 0.000 |
| IBIL | 19331 | 1.018 | [1.010,1.027] | 0.000 |
| TBA | 19330 | 1.239 | [1.215,1.264] | 0.000 |
| TC | 18318 | 2.278 | [2.178,2.384] | 0.000 |
| GLU | 14686 | 3.450 | [1.917,6.208] | 0.000 |
| Gender |  |  |  |  |
| Female | 8375 |  |  |  |
| Male | 11613 | 2.655 | [2.474,2.851] | 0.000 |
| Age |  |  |  |  |
| ＜60 | 9507 |  |  |  |
| ≥60 | 10481 | 11.205 | [10.166,12.349] | 0.000 |

**Abbreviations: AST**: Aspartate aminotransferase. **ALT**: Alanine aminotransferase. **GGT**: Gamma-glutamyl transferase. **ALP**: Alkaline phosphatase. **ChE**: Cholinesterase. **TP**: Total protein. **ALB**: Albumin. **GLO**: Globulin. **A/G**: Albumin/Globulin ratio. **TBIL**: Total bilirubin. **DBIL**: Direct bilirubin. **IBIL**: Indirect bilirubin. **TBA**: Total biliary acid. **TC**: Total cholesterol. **GLU**: Glucose. **OR**: odds ratio. **95% CI**: 95% confidence interval.

Table S9. One-way regression analysis of cohort 2 samples

| **name** | **N** | **OR** | **95%CI** | **P-value** | **N'** | **OR'** | **95%CI'** | **P-value'** |
| --- | --- | --- | --- | --- | --- | --- | --- | --- |
| ALT | 1157 | 1.041 | [1.027,1.056] | 0.000 | 1003 | 1.037 | [1.018,1.057] | 0.000 |
| ALP | 1157 | 1.040 | [1.033,1.047] | 0.000 | 1003 | 1.038 | [1.030,1.046] | 0.000 |
| AST | 1153 | 1.043 | [1.024,1.061] | 0.000 | 1001 | 1.063 | [1.038,1.090] | 0.000 |
| GGT | 1157 | 1.064 | [1.051,1.077] | 0.000 | 1003 | 1.054 | [1.039,1.069] | 0.000 |
| TBIL | 1157 | 5.068 | [3.015,8.520] | 0.000 | 1003 | 4.900 | [2.584,9.290] | 0.000 |
| TP | 1157 | 0.759 | [0.578,0.998] | 0.049 | 1003 | 0.898 | [0.632,1.277] | 0.549 |
| ALB | 1157 | 0.057 | [0.036,0.090] | 0.000 | 1003 | 0.094 | [0.054,0.165] | 0.000 |
| GLO | 1157 | 3.913 | [2.865,5.346] | 0.000 | 1003 | 3.256 | [2.177,4.870] | 0.000 |
| A/G | 1157 | 0.039 | [0.022,0.070] | 0.000 | 1003 | 0.071 | [0.035,0.146] | 0.000 |
| TC | 1157 | 1.003 | [0.999,1.007] | 0.126 | 1003 | 1.009 | [1.004,1.013] | 0.001 |
| TG | 1157 | 1.026 | [1.022,1.030] | 0.000 | 1003 | 1.022 | [1.017,1.026] | 0.000 |
| HDL-C | 1157 | 0.959 | [0.950,0.968] | 0.000 | 1003 | 0.978 | [0.967,0.989] | 0.000 |
| GLU | 1157 | 1.184 | [1.157,1.212] | 0.000 | 1003 | 1.180 | [1.149,1.213] | 0.000 |
| Age |  |  |  |  |  |  |  |  |
| ＜60 | 657 |  |  |  | 534 |  |  |  |
| ≥60 | 500 | 7.994 | [6.127,10.431] | 0.000 | 424 | 4.363 | [3.161,6.022] | 0.000 |
| Gender |  |  |  |  |  |  |  |  |
| Male | 586 |  |  |  | 534 |  |  |  |
| Female | 571 | 0.740 | [0.585,0.937] | 0.012 | 469 | 0.917 | [0.672,1.251] | 0.584 |

**Abbreviations: AST:** Aspartate aminotransferase. **ALT:** Alanine aminotransferase. **GGT:** Gamma-glutamyl transferase. **ALP:** Alkaline phosphatase. **ChE:** Cholinesterase. **TP:** Total protein. **ALB:** Albumin. **GLO:** Globulin. **A/G:** Albumin/Globulin ratio. **TBIL:** Total bilirubin. **DBIL:** Direct bilirubin. **IBIL:** Indirect bilirubin. **TBA:** Total biliary acid. **TC:** Total cholesterol. **TG:** Triglyceride. **HDL-C:** High-density lipoprotein cholesterol. **GLU:** Glucose. **NA:** indicates that data for this indicator are indeed available in the database. **Var:** variable. **OR:** odds ratio. **95% CI:** 95% confidence interval. In Cohort 2, the N' group adjusted for ASCVD prevalence based on BMI, recent alcohol use, diabetes, hypertension. **OR':** odds ratio-adjusted. **95% CI':** 95% confidence interval-adjusted.

Table S10. Sensitivity analysis (E-value) for liver function indicator in the subgroup analysis

| **Cohort 1** | | |  | **Cohort 2** | | | | |
| --- | --- | --- | --- | --- | --- | --- | --- | --- |
| **Var** | **OR(95%Cl)** | **E** |  | **Var** | **OR(95%Cl)** | **E** | **OR**'(**95%Cl**') | **E**' |
| **AST** |  |  |  | **ALT** |  |  |  |  |
| Total | 1.064(1.059, 1.069) | 1.32 |  | Total | 1.041(1.027, 1.056) | 1.25 | 1.037(1.018, 1.057) | 1.23 |
| ＜60 | 1.094(1.086, 1.102) | 1.41 |  | ＜60 | 1.063(1.040, 1.086) | 1.32 | 1.041(1.012, 1.072) | 1.25 |
| ≥60 | 1.024(1.017, 1.032) | 1.18 |  | ≥60 | 1.023(1.001, 1.045) | 1.18 | 1.028(1.001, 1.056) | 1.20 |
| Male | 1.041(1.034, 1.047) | 1.25 |  | Male | 1.024(1.007, 1.042) | 1.18 | 1.027(1.004, 1.051) | 1.19 |
| Female | 1.082(1.073, 1.090) | 1.38 |  | Female | 1.068(1.041, 1.097) | 1.34 | 1.067(1.028, 1.107) | 1.33 |
| **ALT** |  |  |  | **ALP** |  |  |  |  |
| Total | 1.045(1.041, 1.048) | 1.26 |  | Total | 1.040(1.033, 1.047) | 1.24 | 1.038(1.030, 1.046) | 1.24 |
| ＜60 | 1.078(1.073, 1.083) | 1.37 |  | ＜60 | 1.048(1.036, 1.059) | 1.27 | 1.043(1.028, 1.057) | 1.25 |
| ≥60 | 1.023(1.016, 1.031) | 1.18 |  | ≥60 | 1.021(1.012, 1.029) | 1.17 | 1.023(1.013, 1.033) | 1.18 |
| Male | 1.022(1.019, 1.026) | 1.17 |  | Male | 1.029(1.020,1.038) | 1.20 | 1.029(1.018, 1.039) | 1.20 |
| Female | 1.060(1.054, 1.067) | 1.31 |  | Female | 1.052(1.042, 1.063) | 1.29 | 1.050(1.037, 1.063) | 1.28 |
| **GGT** |  |  |  | **AST** |  |  |  |  |
| Total | 1.056(1.053, 1.060) | 1.30 |  | Total | 1.043(1.024, 1.061) | 1.25 | 1.063(1.038, 1.090) | 1.32 |
| ＜60 | 1.076(1.071, 1.080) | 1.36 |  | ＜60 | 1.056(1.026, 1.086) | 1.30 | 1.060(1.021, 1.101) | 1.31 |
| ≥60 | 1.032(1.025, 1.039) | 1.21 |  | ≥60 | 1.018(0.933, 1.044) | — | 1.044(1.009, 1.080) | 1.26 |
| Male | 1.026(1.023, 1.030) | 1.19 |  | Male | 1.023(1.001, 1.046) | 1.18 | 1.038(1.007, 1.069) | 1.24 |
| Female | 1.096(1.089, 1.103) | 1.42 |  | Female | 1.065(1.034, 1.097) | 1.33 | 1.112(1.063, 1.164) | 1.46 |
| **ALP** |  |  |  | **GGT** |  |  |  |  |
| Total | 1.028(1.027, 1.030) | 1.20 |  | Total | 1.064(1.051, 1.077) | 1.32 | 1.054(1.039, 1.069) | 1.29 |
| ＜60 | 1.033(1.030, 1.035) | 1.22 |  | ＜60 | 1.076(1.057, 1.095) | 1.36 | 1.055(1.031, 1.079) | 1.30 |
| ≥60 | 1.011(1.007, 1.015) | 1.12 |  | ≥60 | 1.045(1.025, 1.065) | 1.26 | 1.045(1.024, 1.067) | 1.26 |
| Male | 1.005(1.003, 1.008) | 1.08 |  | Male | 1.040(1.026, 1.055) | 1.24 | 1.043(1.026, 1.060) | 1.25 |
| Female | 1.056(1.053, 1.060) | 1.30 |  | Female | 1.118(1.090, 1.146) | 1.48 | 1.093(1.060, 1.126) | 1.41 |
| **TBIL** |  |  |  | **GLO** |  |  |  |  |
| Total | 1.029(1.022, 1.036) | 1.20 |  | Total | 3.913(2.865, 5.346) | 7.29 | 3.256(2.177, 4.870) | 5.97 |
| ＜60 | 1.044(1.035, 1.052) | 1.26 |  | ＜60 | 9.214(5.076, 16.725) | 17.91 | 5.046(2.392, 10.643) | 9.56 |
| ≥60 | 1.000(0.987, 1.041) | — |  | ≥60 | 3.696(2.307, 5.923) | 6.85 | 3.438(1.984, 5.957) | 6.33 |
| Male | 1.008(0.999, 1.061) | — |  | Male | 4.614(2.969, 7.171) | 8.70 | 4.476(2.612, 7.670) | 8.42 |
| Female | 1.009(0.988, 1.019) | — |  | Female | 3.524(2.251, 5.519) | 6.51 | 2.231(1.188, 4.192) | 3.89 |
| **DBIL** |  |  |  | **TG** |  |  |  |  |
| Total | 1.527(1.466, 1.592) | 2.42 |  | Total | 1.026(1.022, 1.030) | 1.19 | 1.022(1.017, 1.026) | 1.17 |
| ＜60 | 1.614(1.533, 1.698) | 2.61 |  | ＜60 | 1.032(1.026, 1.039) | 1.21 | 1.030(1.022, 1.038) | 1.21 |
| ≥60 | 1.325(1.201, 1.462) | 1.98 |  | ≥60 | 1.015(1.010, 1.020) | 1.14 | 1.012(1.007, 1.018) | 1.12 |
| Male | 1.393(1.315, 1.475) | 2.13 |  | Male | 1.022(1.017, 1.027) | 1.17 | 1.019(1.013, 1.025) | 1.16 |
| Female | 1.326(1.249, 1.407) | 1.98 |  | Female | 1.029(1.023, 1.035) | 1.20 | 1.025(1.018, 1.033) | 1.19 |
| **IBIL** |  |  |  | **TBIL** |  |  |  |  |
| Total | 1.018(1.010, 1.027) | 1.15 |  | Total | 5.068(3.015, 8.520) | 9.61 | 4.900(2.584, 9.290) | 9.27 |
| ＜60 | 1.039(1.029, 1.049) | 1.24 |  | ＜60 | 2.176(1.030, 4.596) | 3.78 | 3.003(1.093, 8.249) | 5.46 |
| ≥60 | 0.984(0.968, 1.000) | — |  | ≥60 | 3.381(1.493, 7.656) | 6.22 | 3.656(1.478, 9.046) | 6.77 |
| Male | 0.994(0.984, 1.004) | — |  | Male | 5.629(2.807, 11.290) | 10.73 | 6.671(2.840, 15.669) | 12.82 |
| Female | 0.994(0.981, 1.006) | — |  | Female | 3.714(1.568, 8.797) | 6.89 | 2.963(1.020, 8.607) | 5.37 |
| **TC** |  |  |  | **GLU** |  |  |  |  |
| Total | 2.278(2.178, 2.384) | 3.98 |  | Total | 1.184(1.157, 1.212) | 1.65 | 1.180(1.149, 1.213) | 1.64 |
| ＜60 | 2.403(2.269, 2.546) | 4.24 |  | ＜60 | 1.187(1.144, 1.231) | 1.66 | 1.191(1.138, 1.247) | 1.67 |
| ≥60 | 1.792(1.629, 1.971) | 2.98 |  | ≥60 | 1.139(1.106, 1.173) | 1.54 | 1.146(1.108, 1.185) | 1.56 |
| Male | 1.758(1.653, 1.870) | 2.91 |  | Male | 1.201(1.159, 1.244) | 1.69 | 1.210(1.161, 1.262) | 1.71 |
| Female | 3.786(3.499, 4.096) | 7.03 |  | Female | 1.172(1.136, 1.208) | 1.62 | 1.170(1.125, 1.216) | 1.62 |
| **TBA** |  |  |  | **ALB** |  |  |  |  |
| Total | 1.239(1.215, 1.264) | 1.78 |  | Total | 0.057(0.036, 0.090) | 34.58 | 0.094(0.054, 0.165) | 20.76 |
| ＜60 | 1.182(1.155, 1.209) | 1.65 |  | ＜60 | 0.031(0.015, 0.065) | 64.01 | 0.088(0.035, 0.218) | 22.22 |
| ≥60 | 1.206(1.148, 1.268) | 1.70 |  | ≥60 | 0.144(0.075, 0.276) | 13.37 | 0.158(0.074, 0.336) | 12.14 |
| Male | 1.188(1.154, 1.223) | 1.66 |  | Male | 0.053(0.028, 0.101) | 37.23 | 0.084(0.040, 0.175) | 23.30 |
| Female | 1.265(1.231, 1.300) | 1.84 |  | Female | 0.036(0.018, 0.073) | 55.05 | 0.075(0.029, 0.192) | 26.16 |
| **GLU** |  |  |  | **A/G** |  |  |  |  |
| Total | 3.450(1.917, 6.208) | 6.36 |  | Total | 0.039(0.022, 0.070) | 50.78 | 0.071(0.035, 0.146) | 27.66 |
| ＜60 | 3.638(1.987, 6.659) | 6.74 |  | ＜60 | 0.006(0.002, 0.019) | 332.83 | 0.028(0.007, 0.110) | 70.93 |
| ≥60 | NA | NA |  | ≥60 | 0.081(0.037, 0.180) | 24.18 | 0.100(0.040, 0.247) | 19.49 |
| Male | NA | NA |  | Male | 0.033(0.015, 0.075) | 60.10 | 0.053(0.021, 0.134) | 37.23 |
| Female | 3.857(2.017, 7.377) | 7.18 |  | Female | 0.031(0.013, 0.076) | 64.01 | 0.084(0.025, 0.279) | 23.30 |
| **TP** |  |  |  | **HDL-C** |  |  |  |  |
| Total | 0.770(0.763, 0.777) | 1.92 |  | Total | 0.959(0.950, 0.968) | 1.25 | 0.978(0.967, 0.989) | 1.17 |
| ＜60 | 0.758(0.749, 0.767) | 1.97 |  | ＜60 | 0.935(0.918, 0.952) | 1.34 | 0.962(0.942, 0.983) | 1.24 |
| ≥60 | 0.814(0.801, 0.828) | 1.76 |  | ≥60 | 0.974(0.961, 0.986) | 1.19 | 0.983(0.969, 0.997) | 1.15 |
| Male | 0.770(0.760, 0.780) | 1.92 |  | Male | 0.951(0.936, 0.965) | 1.28 | 0.973(0.955, 0.990) | 1.20 |
| Female | 0.769(0.760, 0.779) | 1.93 |  | Female | 0.964(0.950, 0.977) | 1.23 | 0.975(0.958, 0.992) | 1.19 |
| **ALB** |  |  |  | **TP** |  |  |  |  |
| Total | 0.642(0.633, 0.652) | 2.49 |  | Total | 0.759(0.578, 0.998) | 1.96 | 0.898(0.632,1.277) | — |
| ＜60 | 0.664(0.652, 0.676) | 2.38 |  | ＜60 | 0.736(0.449, 1.205) | — | 1.077(0.578,2.005) | — |
| ≥60 | 0.689(0.669, 0.708) | 2.26 |  | ≥60 | 1.316(0.885, 1.975) | — | 1.31(0.815,2.106) | — |
| Male | 0.637(0.623, 0.651) | 2.52 |  | Male | 0.928(0.636, 1.354) | — | 1.118(0.698,1.792) | — |
| Female | 0.586(0.572, 0.600) | 2.80 |  | Female | 0.564(0.376, 0.847) | 2.94 | 0.664(0.387,1.141) | — |
| **GLO** |  |  |  | **TC** |  |  |  |  |
| Total | 0.884(0.876, 0.892) | 1.52 |  | Total | 1.003(0.999, 1.007) | — | 1.009(1.004, 1.013) | 1.10 |
| ＜60 | 0.824(0.813, 0.835) | 1.72 |  | ＜60 | 1.021(1.014, 1.029) | 1.17 | 1.024(1.014, 1.035) | 1.18 |
| ≥60 | 0.896(0.880, 0.913) | 1.48 |  | ≥60 | 0.997(0.992, 1.002) | — | 1.000(0.994, 1.006) | — |
| Male | 0.888(0.876, 0.900) | 1.50 |  | Male | 0.996(0.991, 1.001) | — | 1.003(0.997,1.010) | — |
| Female | 0.908(0.897, 0.920) | 1.44 |  | Female | 1.012(1.006, 1.017) | 1.12 | 1.016(1.008, 1.024) | 1.14 |
| **A/G** |  |  |  |  |  |  |  |  |
| Total | 0.206(0.180, 0.237) | 9.18 |  |  |  |  |  |  |
| ＜60 | 0.603(0.505, 0.719) | 2.70 |  |  |  |  |  |  |
| ≥60 | 0.302(0.216, 0.421) | 6.08 |  |  |  |  |  |  |
| Male | 0.167(0.136, 0.205) | 11.45 |  |  |  |  |  |  |
| Female | 0.071(0.057, 0.089) | 27.66 |  |  |  |  |  |  |

**Abbreviations: AST:** Aspartate aminotransferase. **ALT:** Alanine aminotransferase. **GGT:** Gamma-glutamyl transferase. **ALP:** Alkaline phosphatase. **ChE:** Cholinesterase. **TP:** Total protein. **ALB:** Albumin. **GLO:** Globulin. **A/G:** Albumin/Globulin ratio. **TBIL:** Total bilirubin. DBIL: Direct bilirubin. **IBIL:** Indirect bilirubin. **TBA:** Total biliary acid. **TC:** Total cholesterol. **TG:** Triglyceride. **HDL-C:** High-density lipoprotein cholesterol. **GLU:** Glucose. **NA:** indicates that data for this indicator are indeed available in the database. **—:** 95 % confidence interval was not statistically significant and did not show E values. **Var:** variable. **OR:** odds ratio. 95% **CI:** 95% confidence interval. **E:**E-value. In Cohort 2, **OR'**, **95% CI'**, and **E'** are odds ratio, 95% confidence intervals, and E values obtained after adjusting for ASCVD prevalence according to body mass index, recent alcohol consumption, diabetes, and hypertension.
